# Can increased leaf photosynthesis be converted into higher crop mass production? A simulation study for rice using the crop model GECROS

**DOI:** 10.1093/jxb/erx085

**Published:** 2017-03-31

**Authors:** Xinyou Yin, Paul C. Struik

**Affiliations:** 1 Centre for Crop Systems Analysis, Department of Plant Sciences, Wageningen University & Research, PO Box 430, 6700 AK Wageningen, The Netherlands

**Keywords:** Crop modelling, crop productivity, GECROS, genetic transformation, photosynthesis, radiation use efficiency, simulation, water use efficiency, yield potential.

## Abstract

Various genetic engineering routes to enhance C_3_ leaf photosynthesis have been proposed to improve crop productivity. However, their potential contribution to crop productivity needs to be assessed under realistic field conditions. Using 31 year weather data, we ran the crop model GECROS for rice in tropical, subtropical, and temperate environments, to evaluate the following routes: (1) improving mesophyll conductance (*g*_m_); (2) improving Rubisco specificity (*S*_c/o_); (3) improving both *g*_m_ and *S*_c/o_; (4) introducing C_4_ biochemistry; (5) introducing C_4_ Kranz anatomy that effectively minimizes CO_2_ leakage; (6) engineering the complete C_4_ mechanism; (7) engineering cyanobacterial bicarbonate transporters; (8) engineering a more elaborate cyanobacterial CO_2_-concentrating mechanism (CCM) with the carboxysome in the chloroplast; and (9) a mechanism that combines the low ATP cost of the cyanobacterial CCM and the high photosynthetic capacity per unit leaf nitrogen. All routes improved crop mass production, but benefits from Routes 1, 2, and 7 were ≤10%. Benefits were higher in the presence than in the absence of drought, and under the present climate than for the climate predicted for 2050. Simulated crop mass differences resulted not only from the increased canopy photosynthesis competence but also from changes in traits such as light interception and crop senescence. The route combinations gave larger effects than the sum of the effects of the single routes, but only Route 9 could bring an advantage of ≥50% under any environmental conditions. To supercharge crop productivity, exploring a combination of routes in improving the CCM, photosynthetic capacity, and quantum efficiency is required.

## Highlight

We model the impact of improving photosynthesis on rice productivity. We consider all major photosynthesis-enhancing approaches under one umbrella and scale them from leaf photosynthesis to crop production.

## Introduction

Yields of major crops have increased steadily during the last decades. Fischer *et al.,* (2014) claimed that, in order to ensure food and energy security for a growing and increasingly demanding population, staple crop production will need to grow by 60% from 2010 to 2050, with the greatest increases in the next 20 years. They further stressed that higher rates of increase than the current rate should be aimed for to drive faster reductions in world hunger and to guard against unanticipated negative contingencies, for example as a result of increasing frequencies of extreme weather under global climate change.

Crop yield per area of land is the production of mass per unit area multiplied by harvest index. Yield gains associated with the first Green Revolution in cereal crops such as wheat and rice were mainly due to increased harvest index by introducing (semi-)dwarfing genes (e.g. Miflin, 2000; Sadras and Lawson, 2011). For further progress to be made, improvement in crop mass production via increasing leaf and canopy photosynthetic capacity and efficiency should be explored (Long *et al.*, 2006; Murchie *et al.*, 2009; Parry *et al.*, 2011; Ort *et al.*, 2015). Evidence suggests that genetic variation in leaf photosynthesis has not been exploited to be incorporated into crop cultivars (e.g. Driever *et al.*, 2014), except for recent releases in which yield gains were accompanied, to some extent, by traits related to increased leaf photosynthesis (Fischer *et al.*, 2014).

Arguably, exploiting natural genetic variation is still the most feasible approach to improve yield traits including photosynthesis (Flood *et al.*, 2011). For example, exploring natural variation in mesophyll conductance for CO_2_ diffusion (*g*_m_) may improve photosynthesis (Gu *et al.*, 2012; Flexas *et al.*, 2013; Chen *et al.*, 2014). However, very often there is little correlation between leaf photosynthesis and crop productivity across germplasm (Driever *et al.*, 2014; Koester *et al.*, 2016) or across individual lines of a segregating population (e.g. Gu *et al.*, 2014a, b), partly because natural variation in leaf photosynthesis and underlying traits is generally small (Driever *et al.*, 2014). To enhance crop productivity at a greater pace, genetic engineering and synthetic biology approaches to improving leaf photosynthesis should be explored (Long *et al.*, 2006, 2015; Singh *et al.*, 2014; Ort *et al.*, 2015; Kromdijk and Long 2016).

Major crops such as rice follow the pathway of C_3_ photosynthesis. Compared with C_4_ crops such as maize, C_3_ crops have lower photosynthetic productivity primarily because ~20–35% of the carbohydrate is lost through photorespiration (Long *et al.*, 2006; Walker *et al.*, 2016), resulting from the oxygenation of ribulose-1,5-biphosphate by Rubisco, the primary enzyme for CO_2_ fixation. Various genetic engineering routes to enhance C_3_ leaf photosynthesis have been proposed to suppress photorespiration, thereby improving crop productivity. These include: replacing Rubisco with foreign forms with higher specificity for CO_2_ relative to O_2_ (*S*_c/o_) (Whitney *et al.*, 2001; Zhu *et al.*, 2004; Parry *et al.*, 2011), designing a photorespiratory bypass (Kebeish *et al.*, 2007), and transforming the C_4_ CO_2_-concentrating mechanism (CCM) (e.g. von Caemmerer *et al.*, 2012) or the cyanobacterial bicarbonate-based CCM (Price *et al.*, 2011, 2013; Lin *et al.*, 2014) into main C_3_ crops.

Some of the engineering approaches have already made progress and were evaluated experimentally for mass production using model plants, such as *Arabidopsis thaliana* engineered with a photorespiratory bypass (Kebeish *et al.*, 2007) and tobacco with a cyanobacterial bicarbonate transporter (Pengelly *et al.*, 2014) and with accelerated recovery from photoprotection (Kromdijk *et al.*, 2016). Any progress for major crops may not be expected in the near future, and modelling should be considered as an important tool to assess the potential of yield improvement by these photosynthesis-enhancing routes. Many researchers (e.g. Zhu *et al.*, 2004; Song *et al.*, 2013; Kromdijk and Long, 2016) have published modelling studies to assess the potential benefit of using various routes in improving photosynthesis; but most of their analyses were based on the simulation of canopy photosynthesis at a fixed leaf area index (LAI) for a given environmental condition.

During the growth cycle of annual field crops, LAI expands initially, reaches its maximum size, and then senesces, and complex interactions and feedback mechanisms can occur between photosynthesis and other physiological components (Yin and Struik, 2008). These complexities should be considered when scaling up from instantaneous leaf assimilation to daily canopy photosynthesis and to total mass production over the growing season (Boote *et al.*, 2013). To that end, Gu *et al.,* (2014*a*) ran numerical simulations using a full crop growth model, GECROS (Yin and van Laar, 2005), and examined the potential of exploiting the natural genetic variation in leaf photosynthesis within a single segregating population for contributing to crop productivity in rice (*Oryza sativa* L.) under field conditions.

Here we ran the crop model GECROS to quantify the extent to which improved leaf photosynthesis, predominantly from genetic engineering to suppress photorespiration, can result in an expected increase in crop mass production under well-watered, as well as water-limited, field conditions, using rice as an example.

## Materials and methods

Model algorithms and approach in applying GECROS (v4.0) for this study are outlined below. Model parameters, if not defined in the text, are given in Table 1.

**Table 1.** Input parameter values for various parts of biochemical leaf photosynthesis models

Category	Symbol	Definition (unit)	C_3_		C_4_	
			Value	Reference	Value	Reference
e^−^ transport	Φ_2LL_	Quantum efficiency of PSII e^−^ transport under limiting light (mol mol^−1^) at *T*_opt_	0.78	Yin *et al.,* (2014)	0.78	Assumed to be the same as for C_3_
	*r*_2/1_	Ratio of Φ_2LL_ to quantum efficiency of PSI e^−^ transport under limiting light (–)	0.85	Genty and Harbinson (1996)	0.85	Assumed to be the same as for C_3_
	θ	Convexity of irradiance response of PSII e^−^ transport rate (–)	0.8	Yin *et al.,* (2009)	0.8	Assumed to be the same as for C_3_
	*f*_cyc_	Fraction of total PSI e^−^ flux that follows cyclic e^−^ transport (–)	0.05	Yin *et al.,* (2006)	0.45^*a*^	Yin and Struik (2012)
	*f*_pseudo_	Fraction of total PSI e^−^ flux that follows pseudocyclic e^−^ transport (–)	0.10	Yin *et al.,* (2006)	0.05	Yin and Struik (2012)
	*f*_Q_	Fraction of total plastoquinone e^−^ flux that follows the Q-cycle (–)	NU	NU	1	Furbank *et al.,* (1990)
	*h*	H^+^ required per ATP production (mol mol^−1^)	NU	NU	4	Yin and Struik (2012)
	α	Fraction of O_2_ evolution in bundle-sheath cells (–)	NA	NA	0.1	Standard value for C_4_ species such as maize
	*x*	Fraction of ATP used for CCM (–)	NA	NA	0.4^*a*^	von Caemmerer and Furbank (1999)
	φ	Extra ATP required for the CCM per CO_2_ fixed (mol mol^−1^)	NA	NA	2^*a*^	von Caemmerer and Furbank (1999)
	*T*_opt_	Optimum temperature for Φ_2LL_ (°C)	23	Data of Yin *et al.,* (2014)	34	Data of Yin *et al.,* (2016)
	Ω	Difference between *T*_opt_ and the temperature at which Φ_2LL_ falls to e^−1^ of its maximum (°C)	36.8	Data of Yin *et al.,* (2014)	38.4	Data of Yin *et al.,* (2016)
Enzyme kinetics and activity	*S*_c/o25_	Relative CO_2_/O_2_ specificity of Rubisco at 25 °C (mol mol^−1^)	3022	Cousins *et al.,* (2010)	2862	Cousins *et al.,* (2010)
	γ_*25_	Half the reciprocal of *S*_c/o25_ (mol mol^−1^)	0.5/*S*_c/o25_	By definition	0.5/*S*_c/o25_	By definition
	*K*_mC25_	Michaelis–Menten constant of Rubisco for CO_2_ at 25 °C (μmol mol^−1^)	291	Cousins *et al.,* (2010)	485	Cousins *et al.,* (2010)
	*K*_mO25_	Michaelis–Menten constant of Rubisco for O_2_ at 25 °C (mmol mol^−1^)	194	Cousins *et al.,* (2010)	146	Cousins *et al.,* (2010)
	χ_Vcmax25_	Linear slope of maximum Rubisco activity at 25°C (*V*_cmax25_) versus (*n*–*n*_b_)^*b*^ (μmol s^−1^ g^−1^)	75	Derived from data of Yin *et al.,* (2009)	93	1.24 times that for C_3_ (Cousins *et al.*, 2010; Perdomo *et al.*, 2015)
	χ_Jmax25_	Linear slope of maximum PSII e^−^ transport rate at 25 °C (*J*_max25_) versus (*n*–*n*_b_) (μmol s^−1^ g^−1^)	100	Harley *et al.,* (1992); Yin *et al.,* (2009)	200	Derived from data of Yin *et al.,* (2011)
	χε_p25_	Linear slope of PEP carboxylation efficiency at 25 °C (ε_p25_) versus (*n*–*n*_b_) (mol s^−1^ g^−1^)	NA	NA	0.791	Derived from data of Yin *et al.,* (2011)
Leaf respiration	*R*_d25_	Day respiration at 25 °C (μmol m^−2^ s^−1^)	0.01*V*_cmax25_	Common assumption	0.01*V*_cmax25_	Assumed to be the same as for C_3_
	*R*_m_	Respiration rate occurring in mesophyll cells (μmol m^−2^ s^−1^)	NA	NA	0.5*R*_d_^*a*^	von Caemmerer and Furbank (1999)
CO_2_ diffusion	*g*_0_	Empirical residual stomatal conductance if light approaches zero (mol m^−2^ s^−1^)	0.01	Leuning (1995)	0.01	Assumed to be the same as for C_3_
	*a*_1_	Empirical constant for *g*_s_ response to VPD (–)	0.9	Derived from Morison and Gifford (1983)	0.9	Set the same as for C_3_ crops^*c*^
	*b*_1_	Empirical constant for *g*_s_ response to VPD (kPa^−1^)	0.15	Derived from Morison and Gifford (1983)	0.15	Set the same as for C_3_ crops^*c*^
	χ_gm25_	Linear slope of mesophyll conductance at 25 °C (*g*_m25_) versus (*n*–*n*_b_) (mol s^−1^ g^−1^)	0.125	Derived from data of Yin *et al.,* (2009); Gu *et al.,* (2012)	NU	NU
	χ_gbs25_	Linear slope of bundle-sheath conductance at 25 °C (*g*_bs25_) versus (*n*–*n*_b_) (mol s^−1^ g^−1^)	NA	NA	0.007^*a*^	Yin *et al.,* (2011)
	*u*_oc25_	Coefficient lumping diffusivities and solubilities of CO_2_ and O_2_ in H_2_O at 25 °C	NA	NA	0.047	von Caemmerer and Furbank (1999)
Temperature response	*E*γ_*_	Activation energy for γ_*_ (J mol^−1^)	24 460	Bernacchi *et al.,* (2002)	27 417	Yin *et al.,* (2016)
	*E*_Vcmax_	Activation energy for *V*_cmax_ (J mol^−1^)	65 330	Bernacchi *et al.,* (2001)	53 400	Yin *et al.,* (2016)
	*E*_KmC_	Activation energy for *K*_mC_ (J mol^−1^)	80 990	Bernacchi *et al.,* (2002)	35 600	Perdomo *et al.,* (2015)
	*E*_KmO_	Activation energy for *K*_mO_ (J mol^−1^)	23 720	Bernacchi *et al.,* (2002)	15 100	Yin *et al.,* (2016)
	*E*_Rd_	Activation energy for *R*_d_ (J mol^−1^)	46 390	Bernacchi *et al.,* (2001)	41 853	Yin *et al.,* (2016)
	*E*_Jmax_	Activation energy for *J*_max_ (J mol^−1^)	88 380^*d*^	Yin and van Laar (2005)	116 439	Yin *et al.,* (2016)
	*D*_Jmax_	Deactivation energy for *J*_max_ (J mol^−1^)	200 000	Harley *et al.,* (1992)	135 982	Yin *et al.,* (2016)
	*S*_Jmax_	Entropy term for *J*_max_ (J K^−1^ mol^−1^)	650	Harley *et al.,* (1992)	458.7	Yin *et al.,* (2016)
	*E*ε_p_	Activation energy for ε_p_ (J mol^−1^)	NA	NA	51 029	Data of Yin *et al.,* (2016)
	*D*ε_p_	Deactivation energy for ε_p_ (J mol^−1^)	NA	NA	130 363	Data of Yin *et al.,* (2016)
	*S*ε_p_	Entropy term for ε_p_ (J K^−1^ mol^−1^)	NA	NA	425.6	Data of Yin *et al.,* (2016)
	*E*_gm_	Activation energy for *g*_m_ (J mol^−1^)	49 600	Bernacchi *et al.,* (2001)	NU	NU
	*D*_gm_	Deactivation energy for *g*_m_ (J mol^−1^)	437 400	Bernacchi *et al.,* (2002)	NU	NU
	*S*_gm_	Entropy term for *g*_m_ (J K^−1^ mol^−1^)	1400	Bernacchi *et al.,* (2002)	NU	NU
	*E*_gbs_	Activation energy for *g*_bs_ (J mol^−1^)	NA	NA	116 767	Yin *et al.,* (2016)
	*D*_gbs_	Deactivation energy for *g*_bs_ (J mol^−1^)	NA	NA	264 604	Yin *et al.,* (2016)
	*S*_gbs_	Entropy term for *g*_bs_ (J K^−1^ mol^−1^)	NA	NA	860	Yin *et al.,* (2016)
	*E*_uoc_	Activation energy for *u*_oc_ (J mol^−1^)	NA	NA	–1630	Yin *et al.,* (2016)
Base leaf N	*n*_b_	Base leaf nitrogen, at and below which leaf photosynthesis is zero (g m^−2^)	0.3	Sinclair and Horie (1989)	0.3	Assumed to be the same as for C_3_

NA, not applicable; NU, not used by the model presented herein.

^*a*^ These parameter values need to be adjusted if the C_4_ model is used for simulating the cyanobacterial CCM (see the text and Table 2).

^*b*^ Where *n* is leaf nitrogen (g N m^−2^); and *n*_b_ is the base leaf nitrogen, below which no leaf photosynthesis is observed.

^*c*^ Data of Morison and Gifford (1983) showed that stomatal sensitivity to VPD could differ between C_3_ and C_4_; such a difference can be mimicked by our stomatal conductance model, Equation 2 for C_3_ and Equation 11 for C_4_ leaves, when using the same values of *a*_1_ and *b*_1_.

^*d*^ Parameter set in GECROS to be dependent on crop species; the value 88 380 was set as default for rice (Yin and van Laar, 2005).

### C_3_ photosynthesis model

The model of Farquhar *et al.,* (1980; the FvCB model hereafter) calculates net CO_2_ assimilation rate (*A*) as the minimum of the Rubisco-limited (*A*_c_) and e^−^ transport-limited (*A*_j_) rates. The two limiting rates can be expressed collectively as:

$$A\;\;=\;\;\frac{(C_{\text{c}}\;\;-\;\;\Gamma_{*})x_{1}}{C_{\text{c}}\;\;+\;\;x_{2}}-R_{\text{d}}$$ (1)

where for *A*_c_, *x*_1_ = *V*_cmax_ and *x*_2_ = *K*_mC_(1 + *O*/*K*_mO_); for *A*_j_, *x*_1_ = [1*–f*_pseudo_/(1*–f*_cyc_)]*J*_2_/4 and *x*_2_ = 2*O* γ _*_, where *x*_1_ is written according to the FvCB model extended by Yin *et al.* (2004) to be compatible with a C_4_ model for which accounting for *f*_cyc_ is required (see later). In the model, *C*_c_ and *O* are the CO_2_ and O_2_ level, respectively, at the carboxylation sites of Rubisco, *J*_2_ is the total PSII e^−^ transport rate, and Γ _*_, defined as *O* γ _*_ (where γ _*_ is half of the inverse of *S*_c/o_) is the CO_2_ compensation point in the absence of day respiration (*R*_d_).

The submodel for stomatal conductance for CO_2_ transfer (*g*_s_) is:

$$g_{\text{s}}=g_{0}+\frac{A\;\;+\;\;R_{\text{d}}}{C_{\text{i}}\;\;-\;\;C_{\text{i*}}}f_{\text{vpd}}$$ (2)

where *g*_0_ is the residual value of *g*_s_ when irradiance approaches zero, *C*_i*_ is the intercellular CO_2_ level (*C*_i_) at which *A* + *R*_d_ = 0, and *f*_vpd_ is the relative effect of leaf-to-air vapour difference (VPD) on *g*_s_ (see later).

CO_2_ transfer from *C*_a_ (the ambient CO_2_ level) to *C*_c_ can be written as (Flexas *et al.*, 2013):

$$C_{\text{i}}\;\;=\;\;C_{\text{a}}\;\;–\;\;A\;\;(\text{1}/g_{\text{b}}\;\;+\;\;\text{1}/g_{\text{s}})$$ (3)

$$C_{\text{c}}\;\;=\;\;C_{\text{i}}\;\;–\;\;A/g_{\text{m}}$$ (4)

Combining Equations 1–4 gives a standard cubic equation, as shown in Supplementary Text 1 at *JXB* online.

### C_4_ photosynthesis model

The C_4_ model of von Caemmerer and Furbank (1999), as modified by Yin and Struik (2009, 2012), is used here. In C_4_ plants, CO_2_ is fixed initially in the mesophyll by phospho*enol*pyruvate (PEP) carboxylase into C_4_ acids that are then decarboxylated to supply CO_2_ to Rubisco, which is localized in the bundle-sheath chloroplasts. The co-ordinated functioning of the ‘Kranz’ anatomy and C_4_ biochemistry enables an effective CCM. The extra ATP consumption for sustaining the CCM requires a higher *f*_cyc_ in C_4_ than in C_3_ photosynthesis (Yin and Struik, 2012; Nakamura *et al.*, 2013). The rate of PEP carboxylation (*V*_p_) could be limited either by the PEP carboxylase or by the rate of e^−^ transport (Yin and Struik, 2009):

$$V_{\text{p}}\;\;=\;\;\text{min}(\varepsilon_{\text{p}}C_{\text{i}},x\;J_{\text{2}}z/\varphi )$$ (5)

where ε_p_ is the initial carboxylation efficiency of the PEP carboxylase, φ is the extra ATP required for the CCM per CO_2_ fixed, and *z* is the conversion factor of *J*_2_ into the ATP production rate: *z* = (2 + *f*_Q_–*f*_cyc_)/[*h*(1–*f*_cyc_)] (here *h* is the H^+^:ATP ratio; Yin *et al.*, 2004; Yin and Struik, 2012), and *x* represents the fraction of ATP partitioned to the reactions associated with the operation of *V*_p_. In the standard C_4_ model for malic-enzyme subtypes such as crop plants maize and sorghum, *x* was set to 0.4, arising from φ/(3 + φ), where φ = 2, and 3 is mol ATP required for the Calvin cycle to fix 1 mol CO_2_.

An effective CCM requires a small bundle-sheath conductance (*g*_bs_) as *g*_bs_ determines the CO_2_ leakage from the bundle sheath to the mesophyll (*L*) that affects CO_2_ assimilation (von Caemmerer and Furbank, 1999):

$$L\;\;=\;\;g_{\text{bs}}\left(C_{\text{c}}\;\;–\;\;C_{\text{i}}\right)$$ (6)

$$A\;\;=\;\;V_{\text{p}}\;\;–\;\;L\;\;–\;\;R_{\text{m}}$$ (7)

Equations 5–7 can be combined to result in:

$$C_{\text{c}}\;\;=\;\;aC_{\text{i}}\;\;+\;\;\left(b\;\;–\;\;A\;\;–\;\;R_{\text{m}}\right)/g_{\text{bs}}$$ (8)

where *a* = 1 + ε_p_/*g*_bs_ and *b* = 0 if *V*_p_ is PEP carboxylase limited, and *a* = 1 and *b* = *xJ*_2_*z*/φ if *V*_p_ is e^−^ transport limited (Yin and Struik, 2009).

The rate of CO_2_ fixation by Rubisco is modelled in the same way as for C_3_ photosynthesis:

$$A\;\;=\;\;\frac{(C_{\text{c}}\;\;-\;\;\gamma_{*}O)x_{\text{1}}}{C_{\text{c}}+x_{2}O\;\;+\;\;x_{3}}-R_{\text{d}}$$ (9)

where *x*_1_ = *V*_cmax_, *x*_2_ = *K*_mC_/*K*_mO_, *x*_3_ = *K*_mC_ for the enzyme (Rubisco)-limited rate, and *x*_1_ = [1*–f*_pseudo_/(1*–f*_cyc_)]*J*_2_/4, *x*_2_ = 2γ_*_, and *x*_3_ = 0 for the e^−^ transport-limited rate. This form of the e^−^ transport-limited rate implies that it is the NADPH supply that causes the e^−^ transport limitation in C_4_ photosynthesis, in comparison with the standard C_4_ model in which the ATP supply was assumed to cause the e^−^ transport limitation (von Caemmerer and Furbank, 1999). Yin and Struik (2012) discussed the rationale that either the ATP- or the NADPH-limited form can be used for modelling C_4_ photosynthesis provided that *f*_cyc_ and *f*_pseudo_ are set as appropriate. We prefer to use the NADPH-limited form here because the ATP-limited form gives *x*_1_ = (1–*x*)*zJ*_2_/3 (Yin *et al.*, 2011), which would predict a monotonic increase in ATP production rate, thus in an e^−^ transport-limited carboxylation rate, with increasing *f*_cyc_. This does not agree with the more efficient CCM in terms of ATP use (e.g. cyanobacterial CCM; Price *et al.*, 2011). Using the NADPH-limited form allows a revised C_4_ model to simulate photosynthesis of other CCM systems (see below) and to be consistent with the C_3_ photosynthesis modelling where the NADPH-limited form is predominantly used.

A relationship for O_2_ partial pressure between the intercellular air space (*O*_i_) and the sites around Rubisco in bundle-sheath cells (*O*) is described as (von Caemmerer and Furbank, 1999):

$$O\;\;=\;\;\alpha A/\left(u_{\text{oc}}g_{\text{bs}}\right)\;\;+\;\;O_{\text{i}}$$ (10)

A model for *g*_s_ of C_4_ leaves was formulated in a way that slightly differed from Equation 2 of the C_3_ counterpart, to solve analytically for *A* in C_4_ photosynthesis (Yin and Struik, 2009):

$$g_{\text{s}}\;\;=\;\;g_{0}+\frac{A\;\;+\;\;R_{\text{d}}}{C_{\text{s}}\;\;-\;\;C_{\text{s*}}}f_{\text{vpd}}$$ (11)

where *C*_s_ is the CO_2_ level at leaf surface, and *C*_s*_ is the *C*_s_-based CO_2_ compensation point in the absence of *R*_d_ and can be calculated as [*g*_bs_γ_*_O_i_–(1 + γ_*_α/*u*_oc_)*R*_d_ + *R*_m_]/(*g*_bs_ + ε_p_) (Yin and Struik, 2009). Equation 11 for C_4_ and Equation 2 for C_3_, although both empirical, can reproduce experimentally observed linear relationships between *A* and *g*_s_ across various levels of irradiance and nutrients (e.g. Wong *et al.*, 1985) (see Supplementary Fig. S1).

Equation 3 also applies to C_4_ photosynthesis. Combining Equations 3 and 8–11 can yield the standard cubic equation that gives the prediction of *A* (Supplementary Text 1).

### Algorithms common to C_3_ and C_4_ photosynthesis

Some common algorithms were used for C_3_ and C_4_ models. First, *J*_2_ is described as a function of absorbed irradiance *I*_abs_ as (Yin *et al.*, 2006; Yin and Struik, 2012):

$$J_{2}\;\;=\;\;\left(\alpha_{\text{2LL}}I_{\text{abs}}\;\;+\;\;J_{\max}\;\;-\;\;\sqrt{{\left(\alpha_{\text{2LL}}I_{\text{abs}}\;\;+\;\;J_{\max}\right)}^{2}\;\;-\;\;4\theta J_{\max}\alpha_{\text{2LL}}I_{\text{abs}}}\right)/(2\theta )$$ (12)

$$\text{with }\alpha_{\text{2LL}}\;\;=\;\;\Phi_{\text{2LL}}(\text{1}\;\;–\;\;f_{\text{cyc}})/(\text{1}\;\;–\;\;f_{\text{cyc}}\;\;+\;\;r_{\text{2}/\text{1}})$$

Equation 12 differs from the equation used in the standard FvCB model, in that *f*_cyc_, Φ_2LL_, and *r*_2/1_ are introduced. We consider Equation 12 as a better choice as it accounts for the decrease of the overall noncyclic e^−^ transport efficiency (α_2LL_) with increasing cyclic e^−^ transport, which runs at a higher rate in cases involving the CCM.

Secondly, in the *g*_s_ model, *f*_vpd_ is the function for the effect of VPD, which may be described phenomenologically as (Yin and Struik, 2009):

$$f_{\text{vpd}}\;\;=\;\;\frac{1}{\frac{1}{\max(a_{1}-b_{1}\cdot VPD,\;0.01)}-1}$$ (13)

where *a*_1_ and *b*_1_ represent the *C*_i_:*C*_a_ ratio in water vapour-saturated air and the slope of the decrease of this ratio with increasing VPD, respectively, if *g*_0_ in Equation 2 or 11 approaches nil.

Thirdly, a number of parameters are related to leaf temperature (*T*_l_), and some of these can be described by the Arrhenius equation normalized with respect to 25 °C:

$$\text{Parameter}\;\;=\;\;{\text{Parameter}}_{\text{25}}\cdot e^{\left(\frac{1}{298}\;-\;\frac{1}{273+T_{\text{l}}}\right)\frac{E}{R}}$$ (14)

where *R* is the universal gas constant (8.314 J K^−1^ mol^−1^). Equation 14 applies to *R*_d_, γ_*_, *V*_cmax_, *K*_mC_, *K*_mO_, and *u*_oc_. The temperature response of *J*_max_, ε_p_, *g*_m_, and *g*_bs_ is described by the modified Arrhenius equation:

$$\text{Parameter}\;\;=\;\;{\text{Parameter}}_{\text{25}}\cdot e^{\left(\frac{1}{298}-\frac{1}{273+T_{\text{l}}}\right)\frac{E}{R}}\cdot \frac{1\;\;+\;\;e^{\left(S-D/298\right)/R}}{1\;\;+\;\;e^{[S-D/(273+T_{\text{l}})]/R}}$$ (15)

Fourthly, the values at 25 °C of parameters *V*_cmax_, *J*_max_, ε_p_, *g*_m_, and *g*_bs_ can be further quantified as a linear function of leaf nitrogen (N) content (*n*) above a certain base value (*n*_b_):

$${\text{Parameter}}_{\text{25}}\;\;=\;\;\chi (n\;\;–\;\;n_{\text{b}})$$ (16)

where χ has different values for different parameters (e.g. Harley *et al.*, 1992; Yin *et al.*, 2011). We estimated χ_Vcmax25_ for C_3_ leaves from existing data and then projected to C_4_ leaves (Table 1), based on the reported higher catalytic turnover rate of C_4_ Rubisco than that of C_3_ Rubisco (Seemann *et al.*, 1984; Sage, 2002; Cousins *et al.*, 2010; Perdomo *et al.*, 2015). There is less information about the difference in χ_Jmax25_ between C_3_ and C_4_ types, but our χ_Jmax25_ estimates (Table 1) are in line with Makino *et al.,* (2003), who reported a considerably higher photosynthetic N use efficiency under saturated CO_2_ conditions in C_4_ than in C_3_ leaves.

Fifthly, experimental evidence suggests that Φ_2LL_ responds to temperature (Bernacchi *et al.*, 2003; Yin *et al.*, 2014). Due to the lack of understanding of this response, we empirically express the factor for the temperature effect, using a normal distribution alike equation (June *et al.*, 2004):

$$F_{T\Phi \text{2LL}}=e^{-{\left[(T_{\text{l}}-T_{\text{opt}})/\Omega \right]}^{2}}$$ (17)

Finally, Equations 14, 15, and 17 require *T*_l_, and *T*_l_ is solved from coupled modelling of leaf photosynthesis and transpiration: the algorithms in Supplementary Text 1 solve *A* and *g*_s_ simultaneously; the obtained *g*_s_ is used as input to the Penman–Monteith equation (Monteith, 1973; Goudriaan and van Laar, 1994) to solve leaf transpiration and *T*_l_. This procedure involves iterations, in which *T*_l_ is initially set to be the same as the air temperature and then the solved *T*_l_ is used for re-calculating *A*, *g*_s_, and leaf transpiration (Yin and van Laar, 2005).

### Revising the C_4_ model for simulating the cyanobacterial CCM

The single-cell C_4_ photosynthesis model of von Caemmerer and Furbank (2003; see also Supplementary Text 2) can be used for simulating cyanobacterial photosynthesis (Price *et al.*, 2011). However, this model is hard to solve once it is coupled to a *g*_s_ model (Equation 2 or 11). We therefore revise the above C_4_ model to simulate the cyanobacterial CCM, based on the model concept of Price *et al.* (2011). These revisions are: (i) set *g*_bs_ to a high value to mimic *g*_ch_ (conductance of the chloroplast envelope to CO_2_); (ii) set *V*_p_ as if it stands for the combined rate of cyanobacterial bicarbonate transporters; (iii) set *R*_m_ = *R*_d_; and (iv) re-estimate *f*_cyc_ and *x*, in view of the fact that extra ATP required for the cyanobacterial CCM also comes from the cyclic e^−^ pathway (Shikanai, 2007). The ATP cost of bicarbonate transport may be lower than that of the C_4_ CCM (Price *et al.*, 2013; Furbank *et al.*, 2015). Two single-gene transporters (BicA and SbtA) that have been well characterized in cyanobacteria are considered here, and Price *et al.* (2011) estimated that the two transporters require 0.25 and 0.50 ATP per transport event, respectively (so, φ in Equation 5 is 0.75). We re-estimated *x* as 0.2 and *f*_cyc_ as 0.18 (Table 1), where 0.2 arises from 0.75/(3 + 0.75), and 0.18 arises from the C_4_ model of Yin and Struik (2009) for the balanced NADPH:ATP ratio assuming *h* = 4. This revised C_4_ model gives simulated rates of *A* virtually identical to the model of Price *et al.,* (2011) using the same set of parameter values (Supplementary Text 2) under normal and elevated [CO_2_] conditions.

### Setting scenarios of improved leaf photosynthesis for simulation

Major routes in enhancing photosynthesis will be examined, except for the photorespiratory bypass. Modelling this bypass would require more complicated algorithms and parameters (von Caemmerer, 2013), which cannot be straightforwardly implemented to simulate field environments where modelling of *g*_s_ is also needed. We examined the impact of improving *g*_m_ (Tholen *et al.*, 2012; Flexas *et al.*, 2013), improving *S*_c/o_ (Zhu *et al.*, 2004; Parry *et al.*, 2011), introducing the C_4_ mechanisms into C_3_ crops (von Caemmerer *et al.*, 2012), and using cyanobacterial bicarbonate transporters and the CCM (Price *et al.*, 2011, 2013). Given that efforts to engineer these routes, especially the latter two, into new crops will most probably make progress step-wise, we propose the following nine routes (Table 2):

**Table 2.** Nine photosynthesis-enhancing routes, the corresponding photosynthesis models, and parameter sets used for simulation in this study

Route	Description	Model	Parameter set
1	Improved mesophyll conductance *g*_m_	C_3_	All C_3_ default parameters in Table 1 but χ_gm25_ = 0.375
2	Improved Rubisco specificity *S*_c/o_^*a*^	C_3_	All C_3_ default parameters in Table 1 but *S*_c/o_ = 4427
3	Improved value for both *g*_m_ and *S*_c/o_	C_3_	All C_3_ default parameters in Table 1 but χ_gm25_ = 0.375 and *S*_c/o_ = 4427
4	C_4_ biochemistry introduced	C_4_	All C_4_ parameters (including χ_Vcmax25_ and χ_Jmax25_^*b*^) in Table 1, but χ_bs25_ = 0.125
5	C_4_ Kranz anatomy introduced effectively to minimize CO_2_ leakage	C_4_	All default C_3_ enzymatic parameters plus necessary C_4_ parameters to run C_4_ model in Table 1, but χ_bs25_ = 0.007
6	Complete C_4_ mechanism engineered	C_4_	All C_4_ parameters in Table 1, including low χ_bs25_ (= 0.007)
7	Only cyanobacterial bicarbonate transporters engineered	C_4_	All C_3_ default parameters plus necessary C_4_ parameters to run C_4_ model in Table 1, but χ_gbs25_ = 0.125, φ = 0.75, *x* = 0.2, *f*_cyc_ = 0.18, and *R*_m_ = *R*_d_
8	More elaborate cyanobacterial CCM added	C_4_	The same as Route 7, but χ_gbs25_ = 0.007
9	Complete cyanobacterial CCM engineered	C_4_	The same as Route 8, but with *χ*_Vcmax25_ = 93 and χ_Jmax25_ = 200^*c*^

^*a*^ This route assumes that crop plants are engineered to have a high *S*_c/o25_ of the non-green alga *Griffithsia monilis* while maintaining a similar Rubisco turnover rate (Whitney *et al.,* 2001); any effect of the trade-off between Rubisco *S*_c/o_ and carboxylase turnover rate was not quantified here, and readers are suggested to refer to Zhu *et al*., (2014) on this effect.

^*b*^ Based on measurements on existing maize and wheat plants, parameters χ_Vcmax25_ and χ_Jmax25_ have higher values in C_4_ than in C_3_ leaves (Table 1), probably reflecting the acclimation of C_4_ enzymatic activities to high a CO_2_ environment within the bundle-sheath compartment. While strictly speaking these higher values cannot be guaranteed for hypothetical C_4_ plants of Route 4 which is not yet incorporated with the full Kranz anatomy, high values of χ_Vcmax25_ and χ_Jmax25_ for maize plants (Table 1) were used here for simulation of Route 4 in order to represent the full package of the C_4_ biochemistry components.

^*c*^ Cyanobacterial Rubisco has a higher carboxylation rate than C_3_ Rubisco (Hanson *et al.*, 2016), allowing a higher investment of nitrogen in other photosynthetic protein components. However, we are not aware of the N cost for e^−^ transport protein components in cyanobacteria for estimating χ_Jmax25_. For simplicity, χ_Vcmax25_ and χ_Jmax25_ for maize plants (Table 1) are used for this route, based on the expectation of engineering cyanobacterial CCM that approaches typical C_4_ photosynthetic capacities (Price *et al.*, 2013).

(1) Improving *g*_m_, where the slope of *g*_m25_ versus leaf N (χ_gm25_; see Equation 16) is set from its default value 0.125 (Table 1) to be three times higher (i.e. 0.375).
(2) Improving *S*_c/o_, where *S*_c/o25_ is set from its C_3_ default value 3022 (Table 1) to 4427, the observed *S*_c/o25_ for the non-green alga *Griffithsia monilis* (Whitney *et al.*, 2001).
(3) Improving *g*_m_ as well as *S*_c/o_, where χ_gm25_ of 0.375 and *S*_c/o25_ of 4427 are combined.
(4) Introducing C_4_ biochemistry, where the C_4_ photosynthesis model is used with C_4_ kinetic constants (Table 1) while setting *g*_bs_ as high as the C_3_ default *g*_m_ (i.e. setting the slope of *g*_bs25_ versus leaf N; χ_gbs25_; see Equation 16) to 0.125.
(5) Making C_4_ Kranz anatomy function effectively to minimize CO_2_ leakage, where the low χ_gbs25_ (0.007) is combined with C_3_ enzyme kinetic constants (Table 1).
(6) Engineering the complete C_4_ mechanism, where C_4_ kinetic constants (Table 1) combined with a low χ_gbs25_ (0.007) is used in the C_4_ photosynthesis model.
(7) Engineering cyanobacterial single-subunit bicarbonate transporters (BicA and SbtA), where the above revised C_4_ model is combined with the default C_3_ parameters with χ_gbs25_ = 0.125 and the revised values for φ, *x*, *R*_m_, and *f*_cyc_ (Table 2).
(8) Adding a more elaborate cyanobacterial CCM, whereby the carboxysome shell proteins are expressed in chloroplasts to enrich the CO_2_ level around Rubisco similar to the level in the C_4_ bundle-sheath compartment. This route assumes that the chloroplastic C_3_ Rubisco can be reorganized into effective carboxysome structures and other requirements for carboxysome to function are optimized (Price *et al.*, 2011). So, the same model and parameter values as for Route 7 are used, except for χ_gbs25_ which is now set to a lower value of 0.007 as for C_4_ bundle-sheath conductance.
(9) A complete cyanobacterial CCM installed. The complete cyanobacterial CCM will require replacement of the C_3_ Rubisco with a cyanobacterial Rubisco in order to take advantage of better kinetic properties in a high-CO_2_ carboxysome (Long *et al.*, 2016). Based on the expectation of engineering the cyanobacterial CCM that approaches photosynthetic capacities typical of C_4_ plants (Price *et al.*, 2013), we used χ_Vcmax25_ and χ_Jmax25_ of C_4_ photosynthesis (Table 1) for this route. So, this route has the low ATP cost of the cyanobacterial CCM as well as a high enzymatic capacity per unit N to mimic the complete cyanobacterial CCM.

Simulation results of all nine routes will be compared with those of the default in which the C_3_ photosynthesis model with the C_3_ parameter values in Table 1 is used.

### Modelling daily canopy photosynthesis and transpiration

In GECROS, instantaneous canopy photosynthesis and transpiration were calculated using the sun/shade model of de Pury and Farquhar (1997), in which the sunlit and shaded portions of the canopy each are considered as a big leaf, and the above leaf-level model is applied. Assuming an exponential profile of leaf N, total photosynthetically active N for each portion was calculated, and N-dependent photosynthetic parameters *V*_cmax_, *J*_max_, *g*_m_, *g*_bs_, and ε_p_ (see Equation 16) were then scaled up accordingly to each portion of the canopy. Instantaneous rates were scaled up to daily total, using the Gaussian integration (Goudriaan, 1986) to account for any asymmetric diurnal courses of radiation and temperature. These approaches for spatial and temporal extensions apply to the case in the absence of water limitation.

In the presence of water limitation, the available water is partitioned between sunlit and shaded leaves according to the relative share of their potential transpiration (*E*_p_) to obtain their actual transpiration (*E*_a_). The diurnal course of available water is assumed to follow that of radiation. Based on the Penman–Monteith equation, the actual transpiration is transformed into the actual level of stomatal resistance to water vapour (*r*_sw,a_) (Yin and van Laar, 2005):

$$r_{\text{sw},\text{a}}\;\;=\;\;\left(E_{\text{p}}\;\;–\;\;E_{\text{a}}\right)\;(sr_{\text{bh}}\;\;+\;\;\gamma r_{\text{bw}})/(\gamma E_{\text{a}})\;\;+\;\;r_{\text{sw},\text{p}}E_{\text{p}}/E_{\text{a}}$$ (18)

where *r*_sw,p_ is the stomatal resistance to water vapour in the absence of water limitation [ = 1/(1.6*g*_s_), where *g*_s_ is solved from the algorithm in Supplementary Text 1]; *r*_bh_ and *r*_bw_ are the boundary-layer resistance to heat and to water vapour, respectively; γ is the psychrometric constant; and *s* is the slope of the saturated vapour pressure as a function of temperature (kPa °C^−1^). The actual *r*_sw,a_ was converted into the actual *g*_s_, which can be used as input to the analytical quadratic model (see Supplementary Text 3) to estimate the instantaneous actual photosynthesis of the sunlit and shaded leaves. The Gaussian integration was again used to obtain the daily total of the actual photosynthesis. Equation 18 suggests that the impact of water deficit is mainly via stomatal conductance; any non-stomatal effect of water deficit is not modelled in GECROS, except when accounting for changes in *T*_l_ under drought.

### Crop simulation approaches

Simulations were conducted for three sites, Los Baños (14°6'N, 121°9'E; the Philippines), Nanjing (32°56'N, 118°59'E; China), and Shizukuishi (39°41'N, 140°57'E; Japan), representing tropical, subtropical, and temperate rice-growing conditions, respectively, using 31 year (1980–2010) baseline weather data and the present atmospheric [CO_2_] of 400 μmol mol^−1^. We also ran the model under the climate scenario for 2050, at which the expected [CO_2_] is ~550 μmol mol^−1^ and air temperature is 2 °C higher than the baseline (Li *et al.*, 2015). As we only examined the impact of changed leaf photosynthesis on crop productivity, we decoupled the GECROS soil module and used only the crop module for simulation to avoid any confounding effects from uncertainties in simulating soil processes. Potential production was simulated by setting the daily water supply to the crop as non-limiting. Water-limited production was simulated by setting the daily available water for evapotranspiration to no more than 50% of seasonal average daily transpiration simulated for the potential production, which was 1.97, 1.63, and 1.34 mm H_2_O d^−1^ for Los Baños, Nanjing, and Shizukuishi, respectively. Daily N supply was set in such a way that the accumulated N uptake by the crop followed the sigmoid curve of Yin *et al.* (2003) and that the total uptake at maturity reached 20 g N m^−2^, equivalent to the N uptake in high-yielding rice experiments (Setter *et al.*, 1994).

Model parameters for phenology were calibrated (Table 3) so that simulated baseline crop duration was in line with that of the standard cultivar at each site (Li *et al.*, 2015). Crop models are less accurate in predicting spikelet number and therefore harvest index than in predicting crop mass (Boote *et al.*, 2013; Li *et al.*, 2015). To minimize the impact of this uncertainty, we used the simulated total shoot mass (excluding dead leaves) at maturity as the proxy for crop productivity. Input parameters were set as the default values of GECROS for rice (Yin and van Laar, 2005) and those relevant to our study are given in Table 3. As C_4_ enzyme kinetic parameters, especially their temperature responses, are less certain than the C_3_ counterparts (Boyd *et al.*, 2015), additional analysis was conducted for C_4_ simulation (Supplementary Text 4). Similarly, because the exact ATP cost of bicarbonate transport is uncertain (Fridlyand *et al.*, 1996; McGrath and Long, 2014), sensitivity analysis was conducted for Route 9 with regard to this cost (Supplementary Text 5). Further details about GECROS are given in Supplementary Text 6, and source codes of the full GECROS model can be obtained upon request.

**Table 3.** Values of some input parameters of the GECROS crop model relevant to this study

Parameter	Definition (unit)	Value
*S*_la_	Specific leaf area constant for newly emerging leaves (m^2^ g^−1^)	0.03
*n*_Rmin_	Base value of root nitrogen concentration (g g^−1^)	0.005
*n*_Smin_	Base value of stem nitrogen concentration (g g^−1^)	0.005
*n*_RV_	Nitrogen concentration in plant reserves (g g^−1^)	0.0015
*S*_W_	Potential weight of a single grain (g)	0.025
*n*_SO_	Potential nitrogen concentration in grains (g g^−1^)	0.0145
*H*_max_	Maximum final plant height (m)	1.0
*TC*_S_	Time constant for senescence (d)	2
*T*_b_	Base temperature for phenology (°C)	8
*T*_o_	Optimum temperature for phenology (°C)	30
*T*_c_	Ceiling temperature for phenology (°C)	42
*m*_V_	Minimum number of days for pre-flowering period (thermal day^*a*^)	70, 85, 48^*b*^
*m*_R_	Minimum number of days for post-flowering period (thermal day)	28, 32, 22^*b*^
STTIME	Starting time of simulation, equivalent to day number (from 1 January) for seedling emergence	10, 145, 125^*b*^

^*a*^ One thermal day is equivalent to one calendar day if the temperature at each moment of the day is always at the optimum.

^*b*^ Values used for Los Baños (the Philippines), Nanjing (China), and Shizukuishi (Japan), respectively. The STTIME value for Los Baños is for the dry season there (which is the season with the high yield potential), and that for Nanjing is for single-cropping rice (that is predominant in the region, compared with the double-cropping rice where rice is planted twice per year).

## Results and Discussion

### Simulated leaf photosynthesis

**Fig. 1.**
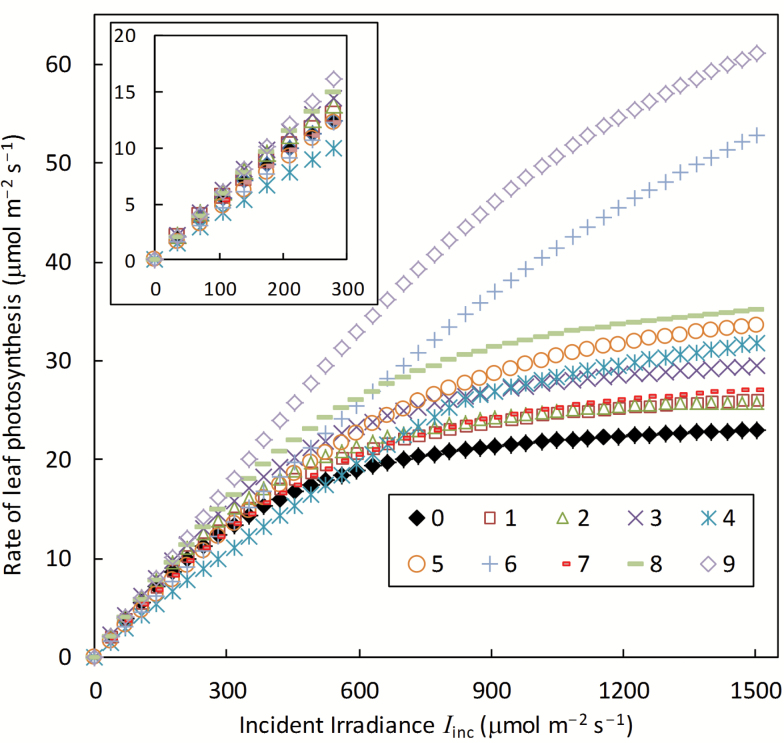
Calculated leaf photosynthesis of the default C_3_ (0) and nine (1–9) photosynthesis-enhancing routes in response to incident irradiance (*I*_inc_). The calculation was made using the model described in the text, based on parameter values listed in Tables 1 and 2 and the following input conditions: *C*_a_ = 400 μmol mol^−1^, *T*_l_ = 25 °C, leaf nitrogen content *n* = 2.3 g m^−2^, *n*_b_ = 0.3 g m^−2^, VPD = 2.0 kPa, and leaf photosynthetic absorptance = 0.85. The inset is for the same response curves when *I*_inc_ is <300 μmol m^−2^ s^−1^.

All routes could increase *A* in the light-saturated region, especially Routes 6 and 9 (Fig. 1). In the light-limited region, the impact of the routes was smaller, and Routes 4, 5, and 6 in fact had a negative effect (see the inset of Fig. 1). This negative impact is associated with the two extra ATPs required for PEP regeneration in the C_4_ cycle (von Caemmerer and Furbank, 1999), for which a high *f*_cyc_ is required (Yin and Struik, 2012; Table 1). In Route 4 where this high ATP cost was not compensated by an effective CCM to suppress photorespiration, the negative effect was particularly high. Because these routes act differently for the light-saturated and limited regions, the curvature in the light response curve was diverse (Fig. 1) despite the same curvature factor θ (0.8; Table 1) used for Equation 12 describing the light response of PSII e^−^ transport rate for all these curves.

### Simulated canopy photosynthesis

**Fig. 2.**
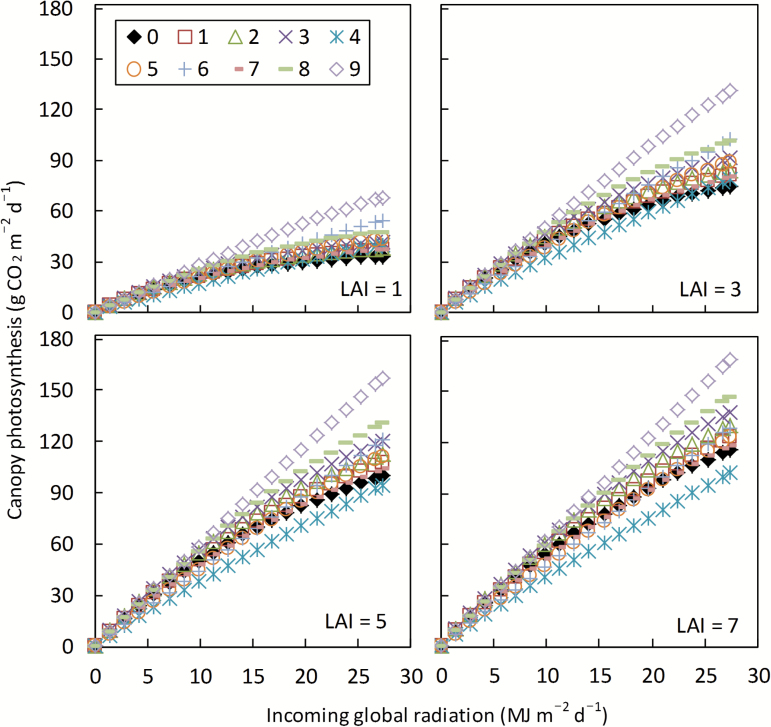
Calculated daily canopy photosynthesis of the default C_3_ (0) and nine (1–9) photosynthesis-enhancing routes in response to daily incoming global solar radiation, for four different sizes of canopy [leaf area index (LAI) = 1, 3, 5, and 7, respectively]. The calculation was made using the model described in the text, based on parameter values listed in Tables 1 and 2 and the following input conditions: *C*_a_ = 400 μmol mol^−1^, *T*_l_ = 25 °C, canopy average leaf nitrogen = 2.3 g m^−2^, *n*_b_ = 0.3 g m^−2^, VPD = 2.0 kPa, daylength = 13 h d^−1^, fraction of diffuse irradiance = 0.2, and canopy average leaf angle (from horizontal) = 65 °. Light extinction coefficient and nitrogen extinction coefficient required for the canopy photosynthesis model were calculated using the formulae as in GECROS with leaf scattering coefficient of 0.2 for PAR.

Not surprisingly, the calculated daily canopy photosynthesis (*A*_canopy,d_) increased with increasing LAI (Fig. 2), due to a higher interception of photosynthetically active radiation (PAR) at higher LAI. Also, the light response curve of *A*_canopy,d_ became increasingly linear with increasing LAI, because at high LAI, leaves in the canopy are predominantly light limited, and within the light-limited range leaf photosynthesis increases almost linearly with light level (Fig. 1). Because the difference in leaf photosynthesis among the routes was mainly recognized in the light-saturated region (Fig. 1), the ratio of *A*_canopy,d_ of photosynthesis-enhancing routes to that of the default C_3_ route increased with increasing radiation level, and decreased with increasing LAI (Fig. 2). *A*_canopy,d_ of Route 4, compared with the default C_3_ route, was notably lower, regardless of the radiation level, when LAI was ≥3 (Fig. 2).

### Default simulation for crop durations and mass production

Using GECROS, we simulated crop duration and mass production. A 2 °C warming for 2050, relative to the present climate, was simulated to shorten crop duration by ~5, 10, and 20 d, for tropical, subtropical, and temperate environments, respectively (Table 4). This different effect across the environments is due to the fact that temperature during the growing season in the tropics is around the optimum value, at which warming is expected to have a smaller effect than at the other sites where the growing season temperature is mostly in the range where development rate increases greatly with warming.

**Table 4.** Days from seedling emergence to flowering and to maturity, aboveground mass at maturity, and season-long canopy photosynthesis and canopy transpiration of rice crop simulated under the default scenario (SDs of the mean of 31 years in parentheses) for the present climate and the 2050 climate, under either potential or water-limited environments, in three representative sites

Site	Los Baños (tropics)				Nanjing (subtropics)				Shizukuishi (temperate)			
Production level	Potential		Water limited		Potential		Water limited		Potential		Water limited	
Climate	Present	2050	Present	2050	Present	2050	Present	2050	Present	2050	Present	2050
Days to flowering	80.6 (1.8)	76.2 (1.2)	–	–	98.3 (2.2)	95.7 (1.0)	–	–	98.7 (5.8)	87.0 (4.4)	–	–
Days to maturity	110.7 (2.0)	105.2 (1.3)	–	–	143.6 (7.6)	133.0 (2.2)	–	–	133.4 (14.8)	113.8 (5.8)	–	–
Crop mass (g DM m^−2^)	1979.9 (55.4)	2141.7 (59.0)	1538.2 (60.4)	1644.8 (69.0)	2246.1 (93.1)	2420.3 (109.5)	1662.4 (108.4)	1782.9 (104.7)	1694.3 (65.5)	1803.4 (73.4)	1193.5 (104.6)	1272.6 (74.9)
Canopy photosynthesis (g CO_2_ m^−2^)	5225.8 (145.7)	5598.9 (157.2)	4408.3 (130.5)	4600.7 (147.2)	5914.8 (231.7)	6330.9 (286.6)	4793.3 (185.9)	5011.2 (169.8)	4510.6 (153.1)	4702.0 (152.8)	3543.9 (218.3)	3668.0 (142.9)
Canopy transpiration (mm H_2_O)	435.7 (25.4)	413.8 (18.9)	177.8 (3.6)	168.1 (3.1)	468.5 (37.3)	470.6 (33.4)	167.1 (4.3)	167.2 (3.2)	357.2 (20.0)	328.2 (19.8)	121.7 (10.3)	109.3 (5.6)
WUE^*b*^ (g m^−2^ mm^−1^)	5.1	6.0	9.7	11.0	5.6	6.1	11.2	11.9	5.3	6.1	11.4	13.4

–, simulations assumed that drought had no impact on phenological development, so the predicted phenology was the same under water-limited as under the potential production level.

^*a*^ Water use efficiency, defined as total crop mass production divided by the amount of water transpired during the growth season.

Despite the shorter duration, simulated aboveground mass at crop maturity increased for 2050 compared with the present climate (Table 4), largely due to CO_2_ elevation from 400 μmol mol^−1^ to 550 μmol mol^−1^. This is because we implicitly assumed that future breeding can develop rice cultivars capable of coping with any effect of warming on spikelet sterility, so the effect of CO_2_ elevation was dominant. However, a recent FACE (free-air CO_2_ enrichment) study (Cai *et al.*, 2016) using a present cultivar showed that yields of rice were decreased by 17–35% under the combination of elevated CO_2_ and temperature, compared with the ambient condition, due to fewer filled grains at the elevated temperature. As expected, water limitation decreased mass production, but increased water use efficiency (WUE) (Table 4). The WUE differed little among the three sites, but was higher for 2050 than for the present climate, partly due to increased *A*_canopy,d_ and partly due to generally decreased canopy transpiration under the 2050 climate (Table 4). The reduced canopy transpiration for the 2050 climate was largely a result of partial stomatal closure induced by higher [CO_2_] (e.g. Wong *et al.*, 1985).

### Impact of photosynthesis-enhancing routes on crop mass production

Compared with the default C_3_ photosynthesis, all routes increased aboveground mass production, except for three cases for the potential production–2050 climate combination where the benefit from Route 5 was virtually nil or slightly negative (Table 5). In general, the benefit from Routes 1, 2, and 7 was ≤10%. All routes resulted in higher benefits in the presence of drought than in the absence of drought, and under the present climate than for 2050. This could be explained by the shape of a diminishing return for *A*–*C*_i_ curves, because drought and the present climate both result in a lower *C*_i_ compared with the potential production level and the 2050 climate, respectively.

**Table 5.** The percentage increase of the 31 year average aboveground mass by nine photosynthesis-enhancing routes, relative to that shown in Table 4 for the default route, in rice crop simulated for the present climate and the 2050 climate, under either potential or water stress environments, in three representative sites

Site		Los Baños (tropics)				Nanjing (subtropics)				Shizukuishi (temperate)			
Production level		Potential		Water limited		Potential		Water limited		Potential		Water limited	
Climate		Present	2050	Present	2050	Present	2050	Present	2050	Present	2050	Present	2050
Route^*a*^	1	4.3	2.5	4.8	3.1	4.2	2.6	4.5	4.1	4.3	2.7	4.5	4.1
	2	8.8	8.0	7.5	6.8	9.3	8.5	11.7	9.7	9.2	8.1	11.0	9.2
	3	12.9	9.9	13.6	12.5	14.0	10.8	16.8	13.8	13.5	10.2	15.5	14.0
	4	10.4	4.1	12.4	6.4	8.0	3.9	11.8	6.2	14.8	8.3	19.2	10.4
	5	7.6	–0.8	26.6	13.6	5.0	–2.4	24.5	11.6	7.0	–0.7	26.6	14.9
	6	38.0	23.1	51.2	33.8	33.0	21.9	50.5	34.1	39.8	25.4	54.5	36.0
	7	5.4	1.6	9.1	5.2	4.5	0.8	10.6	6.0	5.5	2.1	11.3	7.7
	8	17.9	10.5	39.7	28.7	18.1	10.7	39.9	27.9	19.1	11.3	38.7	28.1
	9	70.1	57.5	78.5	61.2	63.2	51.3	74.8	57.9	60.8	49.0	73.8	57.4

^*a*^ Route numbers correspond to those defined in Table 2.

Route combinations had an equal effect to, or a larger effect than, the sum of the routes acting alone. For example, the benefit from Route 3 (improving both *g*_m_ and *S*_c/o_) was about the sum of the benefits from Route 1 (improving *g*_m_) and Route 2 (improving *S*_c/o_) for the potential production and was higher than the sum of the two for the water-limited condition (Table 5).

The benefit from Route 6 (the complete C_4_ mechanism) was considerably higher than the sum of the benefits from Route 4 (C_4_ biochemistry components) and Route 5 (Kranz anatomy components for low *g*_bs_) for any condition (Table 5). This result suggests that the ongoing programme of installing C_4_ photosynthesis into C_3_ crops (von Caemmerer *et al.*, 2012), if successful, needs to engineer the complete C_4_ mechanism. The benefit of a partial engineering is only marginal or even counter-productive under future high-CO_2_ environments (see Route 5 in Table 5), because of the high ATP cost for operating the C_4_ cycle. In an earlier preliminary simulation analysis (Yin and Struik, 2008), we showed that a low *g*_bs_ alone would increase rice yield in the tropics by ~25%. However, that analysis used an arbitrarily low *g*_bs_ and a version of the C_4_ model that assumes an H^+^:ATP ratio of 3, whereas a recent analysis suggested that this ratio is most probably 4 (Yin and Struik, 2012), suggesting that the model version Yin and Struik (2008) used may have underestimated quantum requirement for the C_4_ CCM. From our present analysis, even with the complete C_4_ mechanism, its advantage over the C_3_ default was simulated to be >50% only under the combination of water limitation and the present climate; for other conditions, its advantage ranged between 22% and 40% (Table 5).

As engineering for the complete Kranz anatomy is challenging, the CCM in cyanobacteria, which is probably less expensive energetically, has been suggested as an obvious alternative to engineer (Price *et al.*, 2011, 2013; Furbank *et al.*, 2015). Our simulation showed that the simplest form for the cyanobacterial CCM with bicarbonate transporters, Route 7, had a marginal advantage (Table 5). Pengelly *et al.,* (2014) showed that tobacco plants transformed with the BicA transporter had no discernible effect on CO_2_ assimilation rates, suggesting that BicA was either not located or not activated correctly. Our simulation (Table 5) showed that a more elaborate cyanobacterial CCM, where the carboxysome shell proteins were expressed to enrich the CO_2_ level around Rubisco in chloroplasts (Route 8), had a higher advantage than the equivalent C_4_ CCM (Route 5), largely because of a lower ATP cost assumed for the cyanobacterial CCM. However, its benefit was lower than from the complete C_4_ mechanism, namely Route 6, which includes the additional mechanism that C_4_ plants have a considerably high carboxylation rate per unit leaf N (Evans and von Caemmerer, 2000; Makino *et al.*, 2003). The complete cyanobacterial mechanism (Route 9), which has the low energy cost for the CCM as well as the high carboxylation and e^–^ transport capacity per unit N (presumably as high as for C_4_ plants), was the only route that could bring an advantage of ≥50% under any environmental conditions (Table 5). However, as the exact ATP cost for the cyanobacterial CCM is uncertain, the simulated benefits of Routes 7–9 should be considered as tentative and their real benefits might be lower (Supplementary Text 5).

### Effects of enhanced leaf photosynthesis on some other crop traits

**Fig. 3.**
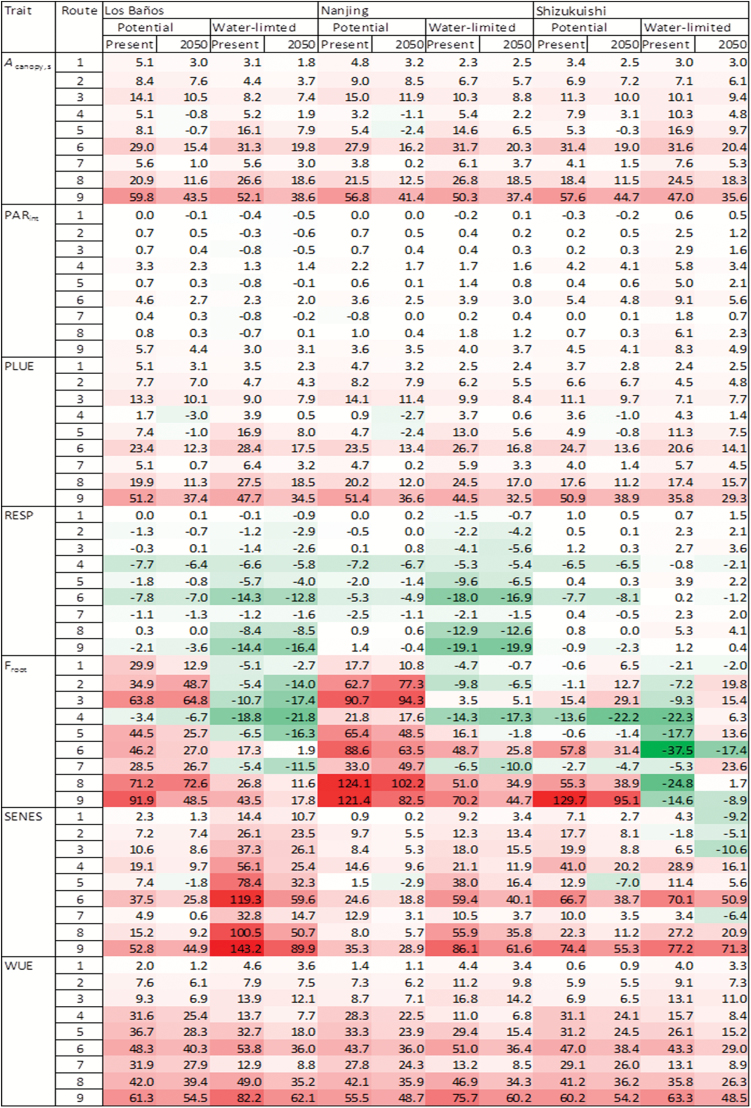
Heat map for the percentage change (%) of the 31 year average trait value for each of the nine photosynthesis-enhancing routes (route numbers defined in Table 2), relative to that for the default route, in a rice crop simulated for the present climate and the 2050 climate, under either potential or water-limited environments, at three representative sites. Traits shown are: *A*_canopy,s_, season-long canopy photosynthesis; PAR_int_, season-long intercepted PAR; PLUE, overall photosynthetic light use efficiency defined as *A*_canopy,s_ divided by PAR_int_; RESP, season-long crop respiration; F_root_, fraction of mass for roots; SENES, shoot mass lost due to leaf senescence; and WUE, water use efficiency. (Colours: white for no change, green for decrease, red for increase, and colour intensity for the magnitude of decrease or increase.)

The benefit from all routes for simulated season-long canopy photosynthesis (*A*_canopy,s_), shown in the upper rows of Fig. 3, differed from that shown in Table 5 for aboveground mass. This difference suggests that other traits were also affected by altered photosynthesis. Aboveground mass can be calculated as:

$$({\text{PAR}}_{\text{int}}\times \text{PLUE}–\text{RESP})\;(\text{1}–{\text{F}}_{\text{root}})-\text{SENES}$$

where PAR_int_ is the season-long intercepted PAR by the crop (MJ m^−2^), PLUE is the overall photosynthetic light use efficiency (= *A*_canopy,s_/PAR_int_, g CO_2_ MJ^−1^ PAR), RESP is season-long crop respiration (g CO_2_ m^−2^), F_root_ is fraction of mass for roots, and SENES is aboveground mass lost after leaf senescence (g DM m^−2^). We obtained the data for these five traits from the GECROS output, and calculated the percentage change of each of these traits for each route relative to the C_3_ default (Fig. 3).

The enhanced leaf photosynthesis from most routes also resulted in increased PAR_int_, although to a small extent only, up to a maximum of 8.3% (Fig. 3). As a result, the percentage increase in PLUE by the routes was generally slightly lower than that in *A*_canopy,s_ (Fig. 3). The increased PAR_int_ stemmed from an increased LAI in the early growth phase (results not shown), in line with the recent result of Kromdijk *et al.,* (2016) showing that increased leaf photosynthesis also resulted in increased leaf area. The increased canopy photosynthesis, on the one hand, increased the component of growth respiration; on the other hand, it decreased the component of maintenance respiration which is modelled in GECROS dependent on crop N status. The net result is that most routes decreased RESP (Fig. 3). The simulated F_root_ generally became higher with photosynthesis-enhancing routes (Fig. 3), as expected from the classical functional equilibrium theory (Brouwer, 1983). However, in the presence of water limitation, F_root_ could be lower compared with the default values, probably because the crop maintained a comparatively high N status under drought. Because a higher photosynthesis resulted in a lower N:C ratio in the crop and GECROS modelled leaf senescence depending on the relative magnitude of N- and C-determined LAI, the most effective enhancing routes, such as Routes 6 and 9, resulted in increased leaf senescence (Fig. 3). This simulation result is in analogy to the faster senescence and reduced LAI in later stages of development for C_3_ crops grown under elevated [CO_2_] in FACE experiments (e.g. Kim *et al.*, 2003). Sinclair *et al.,* (2004) simulated that a 33% increase in leaf photosynthesis may translate into only a 5% increase in soybean grain yield, or a –6% change in grain yield in the absence of additional N, presumably associated with more leaf senescence. Long *et al.,* (2006) reported experimentally a lower than expected crop yield stimulation with rising [CO_2_].

Next, we evaluated the extent to which these secondary effects also contributed to differences in the simulated aboveground mass. This was done from the difference in the significance level of individual terms of multiple linear regression of aboveground mass versus PAR_int_, PLUE, RESP, F_root_, and SENES (Table 6). Although the decreasing effect of SENES on mass could not be identified (because of the collinearity between mass and SENES), the significant effect of the other four terms (PLUE, PAR_int_, RESP, and F_root_) was well estimated. While the primary PLUE had the strongest effect under both potential and water-limited conditions, the secondary PAR_int_ always had the second strongest effect. These results on the importance of the secondary effects on crop-level traits suggest that most existing simulation studies on assessing the impact of engineering photosynthetic targets are incomplete, because computation was only done for leaf photosynthesis (e.g. McGrath and Long, 2014) or for canopy photosynthesis (Zhu *et al.*, 2004; Song *et al.*, 2013).

**Table 6.** The coefficients (with probability of significance in parentheses) of linear regression of 31 year average simulated aboveground mass against the simulated values of five component traits, for either potential or water-limited environments, or using the pooled data for the two environments The five component traits are: PAR_int_, season-long intercepted PAR; PLUE, overall photosynthetic light use efficiency defined as season-long canopy photosynthesis divided by PAR_int_; RESP, season-long crop respiration; F_root_, fraction of mass for roots; SENES, aboveground mass lost due to leaf senescence. Linear regression is given as: *Y* = *b*_0_ + *b*_1_∙*PAR*_*int*_ + *b*_2_∙*PLUE* + *b*_3_∙*RESP* + *b*_4_∙*F*_*root*_ + *b*_5_∙*SENES*

Coefficient (unit)	Potential	Water-limited	Pooled data
*b*_0_ (g DM m^−2^)	–3435.24 (5.92 × 10^–18^)	–1907.22 (5.34 × 10^–32^)	–1751.43 (1.57 × 10^–34^)
*b*_1_ (g DM MJ^−1^ PAR)	4.495 (2.00 × 10^–24^)	3.064 (7.19 × 10^–40^)	3.342 (9.92 × 10^–42^)
*b*_2_ [g DM m^−2^ (g CO_2_ MJ^−1^ PAR)^−1^]	415.65 (9.36 × 10^–35^)	339.49 (3.47 × 10^–48^)	328.83 (1.32 × 10^–70^)
*b*_3_ [g DM (g CO_2_)^−1^]	–0.2635 (0.045)	–0.5735 (6.53 × 10^–22^)	-0.7445 (1.22 × 10^–23^)
*b*_4_ (g DM m^−2^)	–3967.04 (5.05 × 10^–13^)	–539.44 (0.001)	-1245.07 (2.92 × 10^–8^)
*b*_5_ (g DM g^−1^ DM)	–1.1938 (0.153)	1.6387 (6.84 × 10^–6^)	1.9219 (0.001)
*R* ^2^	0.992	0.999	0.993
Data points	60	60	120

### Assessing the importance of individual biochemical targets

While secondary traits were affected, after all one would assess how the primary PLUE is affected by individual biochemical targets or parameters of photosynthesis. We regressed PLUE against individual photosynthetic parameters, with site included as covariate in the regression to remove any effect of possible site differences.

The regression analysis based on simulation results using the C_3_ model (Routes 1–3 plus the default) indicated that manipulating *S*_c/o_ affected PLUE more than manipulating *g*_m_ (results not shown), consistent with the result that the percentage change in PLUE by Route 2 was higher than that by Route 1 (Fig. 3). However, the relative impact of manipulating *S*_c/o_ and *g*_m_ depends on the extent to which they could actually be changed. Furthermore, an improvement in *S*_c/o_ may be at the cost of decreasing *V*_cmax_, because of the often observed negative correlation between Rubisco *S*_c/o_ and carboxylase turnover rate (e.g. Kubien *et al.*, 2008; Perdomo *et al.*, 2015). This negative correlation was not considered here, in view of the fact that the non-green alga *G. monilis* has a high *S*_c/o25_ while maintaining a Rubisco turnover rate similar to C_3_ plants (Whitney *et al.*, 2001). The impact of the trade-off between Rubisco *S*_c/o_ and carboxylase turnover on canopy photosynthesis was analysed by Zhu *et al.,* (2004).

Our simulations using the C_4_ model (Routes 4–9) involved changes in values of a set of parameters. The most important ones are χ_gbs25_ (which determines the effectiveness of the CCM), φ (extra ATP requirement for the CCM, which determines the required *f*_cyc_ and, therefore, light-limited photosynthetic efficiency), and χ_Vcmax25_ or χ_Jmax25_ (which determine light-saturated photosynthetic capacity). Other parameters (e.g. some C_3_ parameters used for Route 5) had little impact on the shape and values of light response curves (results not shown). We therefore conducted the analysis of regressing PLUE versus χ_gbs25_, 3 + φ (total ATP requirement per mol CO_2_ assimilated, ATP_req_), and χ_Jmax25_ (Table 7). There was little effect of sites on PLUE. All three parameters were important for any production level–climate combination. Comparatively, the CCM parameter χ_gbs25_ became most important for water-limited production, because a more effective CCM to elevate the CO_2_ level around Rubisco can more effectively overcome the negative effect of low *C*_i_ under drought. Under the potential production, especially combined with high [CO_2_] of the 2050 climate, the photosynthetic capacity parameter χ_Jmax25_ and quantum efficiency parameter ATP_req_ were comparatively more important (Table 7). These results suggest that photosynthetic capacity, quantum efficiency, and CCM strength all need improving in order to turbocharge canopy photosynthesis. Based on natural variation of leaf photosynthesis, Gu *et al.,* (2014*a*) showed that quantum efficiency parameters had even higher effects than capacity parameters on rice productivity.

**Table 7.** The coefficients with their probability of significance of linear regression of 31 year average simulated PLUE (overall photosynthetic light-use efficiency as defined in Table 6) against three biochemical parameters (χ_gbs25_, ATP_req_, and χ_Jmax25_, representing the strength of the CCM, quantum requirement, and photosynthetic capacity, respectively) used in the C_4_ photosynthesis model, for four cases where potential or water-limited environments were combined with present or 2050 climate conditions

	Potential level				Water-limited level			
	Present climate		2050 climate		Present climate		2050 climate	
	Coefficient	Probability	Coefficient	Probability	Coefficient	Probability	Coefficient	Probability
Intercept	11.674	1.45 × 10^–11^	12.108	2.67 × 10^–12^	9.019	2.31 × 10^–12^	9.385	1.49 × 10^–14^
Nanjing^*a*^	0.097	0.56	0.102	0.50	–0.078	0.48	–0.112	0.15
Shizukuishi^*a*^	0.167	0.32	0.415	0.01	–0.200	0.09	–0.051	0.49
χ_gbs25_	–12.751	1.64 × 10^–7^	–10.574	3.91 × 10^–7^	–10.262	1.84 × 10^–8^	–8.406	2.23 × 10^–9^
ATP_req_^*b*^	–1.237	1.22 × 10^–7^	–1.244	3.46 × 10^–8^	–0.659	1.24 × 10^–6^	–0.676	1.40 × 10^–8^
χ_Jmax25_	0.0162	7.37 × 10^–8^	0.0150	5.12 × 10^–8^	0.0083	1.22 × 10^–6^	0.0072	7.76 × 10^–8^
*R* ^2^	0.963		0.965		0.961		0.976	
Data points	18		18		18		18	

^*a*^ Site was included as the covariate in regression, with Los Baños as the reference having a coefficient of zero.

^*b*^ Total ATP requirement per CO_2_ assimilated (= 3 + φ), i.e. 5 for C_4_ photosynthesis and 3.75 for cyanobacterial photosynthesis (see the text).

## Concluding remarks

We simulated the likely impact of major routes in ongoing programmes using transgenic technology to improve photosynthesis (Table 2). Our analysis showed that improving leaf photosynthesis can result in an increased rice mass production to a different extent (Table 5), thereby also resulting in different improvements in resource use efficiency such as WUE (Fig. 3). However, to supercharge photosynthesis significantly, engineering for a single improvement route can hardly be effective. Some single routes may be counter-productive at the canopy level. For example, installing C_4_ biochemistry, if not combined with an effective CCM, is only beneficial for upper leaves of the canopy, while it has no or even a negative impact for lower shaded leaves because such a mechanism requires extra ATP for the C_4_ cycle. Note that the standard C_4_ model of von Caemmerer and Furbank (1999) for e^−^ transport limitation does not explicitly consider the increased cyclic e^−^ transport due to the extra ATP costs relative to C_3_ photosynthesis, and, therefore, cannot recognize the little advantage of C_4_ photosynthesis under shade. Similarly, the simulation by McGrath and Long (2014) in assessing the potential of cyanobacterial CCM took no account of the extra ATP required by bicarbonate transporters. Our simulation also showed that manipulating photosynthesis may result in unwanted secondary effects on some traits at crop level (e.g. inducing faster senescence if nutrient uptake is not increased). Therefore, the beneficial effect of the single route for high photosynthesis on increasing crop productivity may have previously been overestimated. To supercharge crop productivity, combined routes for improved CCM, photosynthetic capacity, and quantum efficiency are required.

## Supplementary Data

Supplementary data are available at *JXB* online.

**Fig. S1.** Simulated versus observed relationships between *A* and *g*_s_.

**Text 1.** Analytical solution to the cubic equation as a result of combined stomatal conductance, CO_2_ diffusion, and biochemical leaf photosynthesis models.

**Text 2.** Revising the C_4_ photosynthesis model for simulating the cyanobacterial CCM.

**Text 3.** Analytical solution to the quadratic equation as a result of combined CO_2_

**Text 4.** Analysis with respect to temperature response parameters of C_4_ enzyme kinetics.

**Text 5.** Sensitivity analysis with respect to ATP cost for the cyanobacterial CCM.

**Text 6.** Description of the crop model GECROS (version 4.0).

## Supplementary Material

Supplementary_figure_S1_S5_Text_S1_S6
