# Supplementary material for: Can increased leaf photosynthesis be converted into higher crop mass production? A simulation study for rice using the crop model GECROS
Source: J Exp Bot. 2017 Mar 31;68(9):2345–60. doi: 10.1093/jxb/erx085 (PMC5447886; doi:10.1093/jxb/erx085)
Supplement: Supplementary_figure_S1_S5_Text_S1_S6 [file erx085_suppl_Supplementary_figure_S1_S5_Text_S1_S6.pdf]

Supplementary Materials

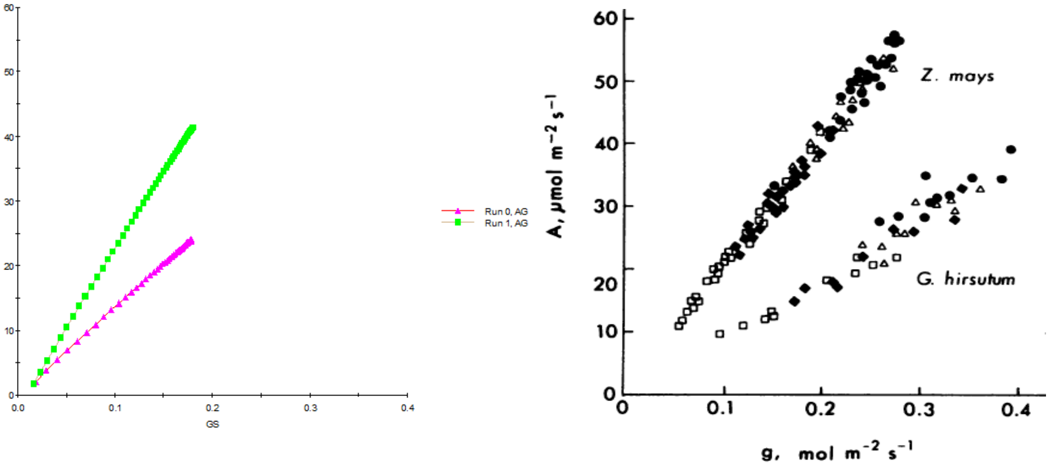

Figure S1 Simulated (left) vs observed (right, from Wong et al. 1985) relationships between *A* (the *y*-axis in μmol m^{-2} s^{-1}) and g_s (the *x*-axis, in mol m^{-2} s^{-1}). The same set of a_1 and b_1 values of eqn (13) (Table 1) was used for simulation of C_3 (pink symbols) and C_4 (green symbols) crops in the left panel. In the right panel, the indicated *G. hirsutum* and *Z. mays* are C_3 and C_4 species, respectively.

Supplementary Text 1

Analytical solution to the cubic equation as result of combined stomatal conductance, CO_2 diffusion and biochemical leaf-photosynthesis models

A general cubic equation is expressed as

$$A^3 + pA^2 + qA + r = 0$$

The solution of this standard form of the cubic equation has three roots:

$$A_1 = -2\sqrt{Q}\cos\left(\frac{\psi}{3}\right) - \frac{p}{3}$$

$$A_2 = -2\sqrt{Q}\cos\left(\frac{\psi + 2\pi}{3}\right) - \frac{p}{3}$$

$$A_3 = -2\sqrt{Q}\cos\left(\frac{\psi + 4\pi}{3}\right) - \frac{p}{3}$$

where

$$Q = (p^2 - 3q)/9$$

$$\psi = \arccos(U/\sqrt{Q^3})$$

$$U = (2p^3 - 9pq + 27r)/54$$

Stomatal conductance and biochemical photosynthesis models can be combined, and ultimately, leaf photosynthesis rate of either C_3 or C_4 type can be expressed in the above cubic equation, where p, q and r are the coefficients that lump individual parameters from the underlying stomatal conductance and biochemical photosynthesis model.

(1) C_3 photosynthesis: The coefficients p, q and r of the general cubic equation for C_3-photosynthesis are:

$$p = -[d + (x_1 - R_d)/g_m + a(1/g_m + 1/g_b) + (g_0/g_m + f_{vpd})c]/m$$

$$q = [d(x_1 - R_d) + ac + (g_0/g_m + f_{vpd})b]/m$$

$$r = -ab/m$$

in which coefficients a, b, c, d and m are expressed as:

$$a = g_0(x_2 + \Gamma_*) + (g_0/g_m + f_{vpd})(x_1 - R_d)$$

$$b = C_a(x_1 - R_d) - \Gamma_* x_1 - R_d x_2$$

$$c = C_a + x_2 + (1/g_m + 1/g_b)(x_1 - R_d)$$

$$d = x_2 + \Gamma_* + (x_1 - R_d)/g_m$$

$$m = 1/g_m + (g_0/g_m + f_{vpd})(1/g_m + 1/g_b)$$

where x_1 and x_2 are defined in the main texts following eqn (1). The root A_1 of the above cubic equation was found to be suitable for calculating either A_c or A_j of C_3 photosynthesis under any combinations of environmental variables C_a, incident irradiance I_{inc}, temperature, and VPD. The minimum of A_c and A_j gives A. Once A is known, it can be used to sequentially solve C_c, C_i and g_s from eqns (1), (4) and (2), respectively.

(2) C_4 photosynthesis: The coefficients p, q and r of the cubic equation for C_4-photosynthesis are:

$$p = [j - (h - lR_d)]/l$$

$$q = (i + jR_d - g)/l$$

$$r = -(f - iR_d)/l$$

in which coefficients *f*, *g*, *h*, *i*, *j* and *l* are expressed as:

$$f = (b - R_{\mathrm{m}} - \gamma_{*} O_{\mathrm{i}} g_{\mathrm{bs}}) x_1 d + a g_{\mathrm{bs}} x_1 C_{\mathrm{a}} d$$

$$g = (b - R_{\mathrm{m}} - \gamma_{*} O_{\mathrm{i}} g_{\mathrm{bs}}) x_1 m - (\alpha\gamma_{*} / u_{\mathrm{oc}} + 1) x_1 d + a g_{\mathrm{bs}} x_1 [C_{\mathrm{a}} m - d / g_{\mathrm{b}} - (C_{\mathrm{a}} - C_{\mathrm{s*}})]$$

$$h = -[(\alpha\gamma_{*} / u_{\mathrm{oc}} + 1) x_1 m + a g_{\mathrm{bs}} x_1 (m - 1) / g_{\mathrm{b}}]$$

$$i = (b - R_{\mathrm{m}} + g_{\mathrm{bs}} x_3 + x_2 g_{\mathrm{bs}} O_{\mathrm{i}}) d + a g_{\mathrm{bs}} C_{\mathrm{a}} d$$

$$j = (b - R_{\mathrm{m}} + g_{\mathrm{bs}} x_3 + x_2 g_{\mathrm{bs}} O_{\mathrm{i}}) m + (\alpha x_2 / u_{\mathrm{oc}} - 1) d + a g_{\mathrm{bs}} [C_{\mathrm{a}} m - d / g_{\mathrm{b}} - (C_{\mathrm{a}} - C_{\mathrm{s*}})]$$

$$l = (\alpha x_2 / u_{\mathrm{oc}} - 1) m - a g_{\mathrm{bs}} (m - 1) / g_{\mathrm{b}}$$

where x_1, x_2 and x_3 are defined in the main text (eqn 9), *a* and *b* are defined in the text below eqn (8), and *d* and *m* are defined as:

$$d = g_0 C_{\mathrm{a}} - g_0 C_{\mathrm{s*}} + f_{\mathrm{vpd}} R_{\mathrm{d}}$$

$$m = f_{\mathrm{vpd}} - g_0 / g_{\mathrm{b}} .$$

The root A_3 of the general cubic equation was found to be suitable for calculating *A* of C_4 photosynthesis under any combination of environmental variables C_{a}, I_{inc}, temperature, and VPD. As either the enzyme activity or the e^- transport can limit both Rubisco and PEP carboxylase reactions, in theory four types of combinations of rate limitations are possible. The minimum of the four rates gives the prediction of *A*. Once *A* is known, g_{s} can be solved from eqn (11), where $C_{\mathrm{s}} = C_{\mathrm{a}} - A/g_{\mathrm{b}}$.

Supplementary Text 2

Revising the C_4-photosynthesis model for simulating the cyanobacterial CCM

Price et al. (2011) used the single-cell C_4 photosynthesis model of von Caemmerer & Furbank (2003) for simulating the cyanobacterial CCM.

In the single-cell C_4 photosynthesis, the initial PEP carboxylation occurs in the mesophyll cytosol. In the chloroplast, the CO_2 released from C_4 acid decarboxylation either is fixed by Rubisco or leaks to the cytosol. Like respiratory CO_2 (R_d), photorespiratory CO_2 (F) is released in the cytosol. So, in analogy to eqn (7) in the main text for the Kranz-anatomy C_4 photosynthesis, net CO_2 assimilation rate in single-cell C_4 photosynthesis can be expressed in terms of the fluxes in cytosol:

$$A = V_p - L - R_m - F$$

where $R_m = R_d$, and F can be expressed as: $F = \Gamma_* x_1 / (C_c + x_2)$ (Farquhar et al. 1980), where x_1 and x_2 are as defined for eqn (1) in the main text. The rate of CO_2 leakage from the chloroplast to the cytosol (L) can be expressed, in analogy to eqn (6):

$$L = g_{ch}(C_c - C_m)$$

where g_{ch} is the chloroplast conductance, and C_m represents the cytosol CO_2 level.

The models for both single-cell and Kranz-anatomy C_4 photosynthesis can be collectively expressed in terms of cytosol CO_2 level (C_m), using the following three equations:

$$A = \frac{(C_c - \Gamma_*)x_1}{C_c + x_2} - R_d$$

$$A = V_p - L - R_m - \zeta F$$

$$L = g_x(C_c - C_m)$$

where for the Kranz-anatomy C_4 photosynthesis: $\zeta = 0$, $g_x = g_{bs}$, and R_m is commonly assumed to be $0.5R_d$; for the single-cell C_4 photosynthesis: $\zeta = 1$, $g_x = g_{ch}$, $R_m = R_d$. The three equations can be combined to solve C_c:

$$C_c = \left(-b + \sqrt{b^2 - 4g_x c}\right) / (2g_x)$$

where $b = x_1 - V_p - R_d + R_m - g_x(C_m - x_2)$, and $c = -(V_p + R_d - R_m + g_x C_m)x_2 - (1-\zeta)\Gamma_* x_1$. Once C_c is solved, it can be used to calculate A from the first equation. The V_p term of the single-cell C_4 model, when applied to model the cyanobacterial CCM, refers to the sum of the individual bicarbonate transport rates (Price et al. 2011).

Simulation using this model showed that although the Kranz C_4 model over-predicts C_c appreciably, it over-predicts the cyanobacterial photosynthesis only by 0.5-2.5% regardless of the value of g_x used, under the present atmospheric CO_2 condition (Fig. S2). Under elevated CO_2, the over-prediction is even smaller. The small over-prediction is expected because C_c is largely determined by g_x and A responds to C_c in a manner of diminishing return. This suggests that the Kranz C_4 photosynthesis model can be effectively used to simulate the impact of introducing the cyanobacterial CCM on crop productivity under the present and elevated atmospheric CO_2 conditions as long as values of parameters φ, x, f_{cyc}, R_m, and g_{bs} are adjusted. However, if g_x is high (e.g. for Route 7 in our analysis) combined for the case of drought where C_m is low, the error can become appreciable.

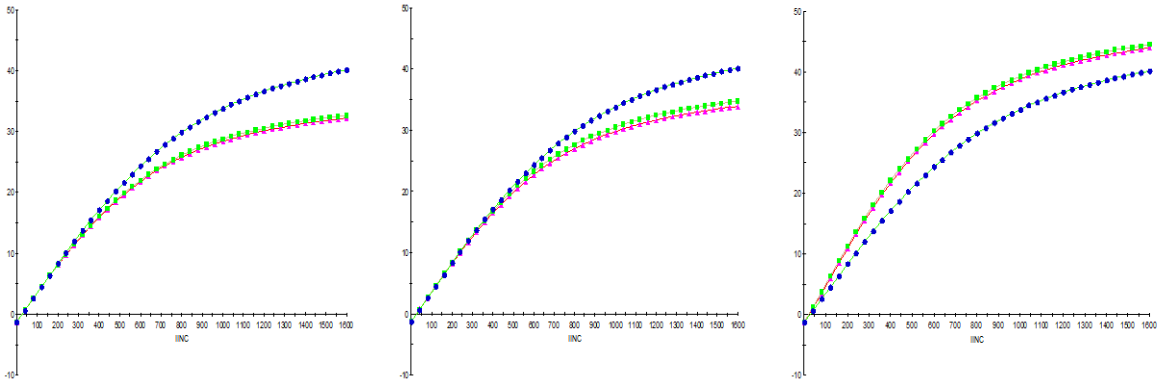

Fig. S2 Simulated rate of photosynthesis (*y*-axis in μmol m^{-2} s^{-1}) in response to incident irradiance I_{inc} (*x*-axis in μmol m^{-2} s^{-1}) in standard Kranz C_4 leaves (blue curve), and in C_3 leaves having cyanobacterial CCM installed with two bicarbonate transporters BicA and SbtA (pink-triangle curve). g_x = 0.45 mol m^{-2} s^{-1} (left panel), or 0.15 mol m^{-2} s^{-1} (middle panel), or 0.003 mol m^{-2} s^{-1} (right panel); other inputs are: C_m = 250 μmol mol^{-1}, R_d = 1.2 μmol m^{-2} s^{-1}, φ = 0.75, x = 0.2 and f_{cyc} = 0.18. The green curve is generated using the Kranz C_4 model using the same set of parameter values (to test if the Kranz C_4 model can be used to simulate photosynthesis installed with the cyanobacterial CCM). The standard Kranz C_4 curve (blue curve) was obtained using g_x = 0.003 mol m^{-2} s^{-1}, φ = 2, x = 0.4 and f_{cyc} = 0.45 (see the main text).

Supplementary Text 3

Analytical solution to the quadratic equation as a result of combined CO_2 diffusion and biochemical photosynthesis models if g_s is known

A general quadratic equation is expressed as

$$pA^2 + qA + r = 0$$

The solution of this standard form of the quadratic equation has two roots:

$$A_1 = \left(-q + \sqrt{q^2 - 4pr}\right)/(2p)$$

$$A_2 = \left(-q - \sqrt{q^2 - 4pr}\right)/(2p)$$

(1) C_3 photosynthesis: Combining eqn (1), (3) and (4) in the main text results in the above general quadratic equation for C_3-photosynthesis, in which the coefficients *p*, *q* and *r* are:

$$p = 1/g_{\mathrm{b}} + 1/g_{\mathrm{s}} + 1/g_{\mathrm{m}}$$

$$q = -p(x_1 - R_{\mathrm{d}}) - (C_{\mathrm{a}} + x_2)$$

$$r = (C_{\mathrm{a}} - \Gamma_*)x_1 - (C_{\mathrm{a}} + x_2)R_{\mathrm{d}}$$

The root A_2 of the general quadratic equation was found to be suitable for calculating *A* of C_3 photosynthesis under any combination of environmental variables.

(2) C_4 photosynthesis: Combining eqns (3) and (8-10) in the main text results in the above general quadratic equation for C_3-photosynthesis, in which the coefficients *p*, *q* and *r* are:

$$p = mg_{\mathrm{bs}} + x_2\alpha / u_{\mathrm{oc}}$$

$$q = t - u + pR_{\mathrm{d}}$$

$$r = tR_{\mathrm{d}} - n$$

where *m*, *n*, *t* and *u* are defined as:

$$m = -a(1/g_{\mathrm{b}} + 1/g_{\mathrm{s}}) - 1/g_{\mathrm{bs}}$$

$$n = (ag_{\mathrm{bs}}C_{\mathrm{a}} + b - R_{\mathrm{m}} - g_{\mathrm{bs}}\gamma_* O_{\mathrm{i}})x_1$$

$$t = ag_{\mathrm{bs}}C_{\mathrm{a}} + b - R_{\mathrm{m}} + g_{\mathrm{bs}}x_3 + g_{\mathrm{bs}}x_2 O_{\mathrm{i}}$$

$$u = (mg_{\mathrm{bs}} - \gamma_* \alpha / u_{\mathrm{oc}})x_1$$

where *a* and *b* are defined in eqn (8). The root A_1 of the general quadratic equation was found to suit for calculating C_4 photosynthesis under any combination of environmental variables.

Supplementary Text 4

Analysis with respect to temperature response parameters of C_4 enzyme kinetics

Our work on predicting the effects of C_3 Rubisco kinetic properties on canopy level photosynthesis and biomass appears well parameterized based on well tested literature data (Table 1), therefore, providing best insights into the likely effects of improving Rubisco and g_m. However, the predictive modelling of installation of a C_4 pathway or a bicarbonate pump are relatively less well supported, due to insufficient information about the kinetic parameters used for C_4 Rubisco, PEP carboxylase and in particular, their temperature responses. Many temperature response parameters for C_4 kinetics shown in Table 1 of the main text were derived by Yin et al. (2016), based on *in vitro* measurements previously reported in the literature across various C_4 species. Only recently did Boyd et al. (2015) report a complete set of C_4 kinetic parameter values in a single species. Some parameter values of Boyd et al. (2015) also have uncertainties; for example, their estimated deactivation energy of V_{pmax} (maximum PEP carboxylation activity) was unusually lower than the estimated activation energy in the modified Arrhenius equation, meaning that the optimum temperature of V_{pmax} cannot be determined. Moreover, they are not for C_4 crop species like maize, but only for *Setaria viridis*. Nevertheless, it is still useful to conduct additional analysis by comparing with this unique set of parameters from a single study in order to assess likely impacts of using a mixing set of C_4 parameter scenarios. Values derived from Boyd et al. (2015) vs those summarised by Yin et al. (2016) are given in Table S1 for temperature response parameters of C_4 kinetics for the model form used in our study. They generate a different photosynthetic response to temperature only when the CO_2 level is low (Fig. S3).

We then conducted a simulation with the full GECROS model, using 31-yr weather data, for Shizukuishi, the site where seasonal temperature fluctuates most among the three, thus also the site for which simulation output is expected to be likely most sensitive to a change in photosynthetic temperature response parameter values. We conducted this simulation for Routes 4 and 6 (Table 2 in the main text), for which the C_4 kinetic parameters are most relevant. It turned out that there were essentially no differences between two sets of simulation using the parameters of Boyd et al. (2015) and of Yin et al. (2016) for any production level-climate scenario combination (Table S2). The little sensitivity to enzyme kinetic temperature response parameters suggests that photosynthesis under field conditions especially for shaded leaves in crop canopies was largely limited by electron transport or that the intercellular level CO_2 level even under water limited conditions was high enough, at least for large parts of a growth season, that a difference in enzyme kinetic parameter values did not lead to a significant change in temperature response curve of leaf photosynthesis (Fig. S3). Therefore, all simulation results in the main text are based on the values of Yin et al. (2016) for these seven parameters.

Table S1 Values of C_4 kinetics derived from Boyd et al. (2015) vs those reviewed by Yin et al. (2016)

Parameter [a]	Unit	Boyd et al. (2015)	Yin et al. (2016) [c]
E_{Vcmax}	J mol^{-1}	78000	53400
E_{KmC}	J mol^{-1}	64200	35600
E_{KmO}	J mol^{-1}	10500	15100
$E_{\gamma*}$	J mol^{-1}	31100	27417
$E_{\varepsilon p}$	J mol^{-1}	58500 [b]	51029
$D_{\varepsilon p}$	J mol^{-1}	73300 [b]	130363
$S_{\varepsilon p}$	J K^{-1} mol^{-1}	250 [b]	425.6

[a] for definitions of the parameters, see Table 1 of the main text;

[b] these parameter values were not directly given by Boyd et al. (2015); they are derived here from equivalent parameter values from Boyd et al (2015) for V_{pmax} and K_p (the Michaelis-Menten constant of the PEP carboxylase) and according to that the initial A-C_i slope $\varepsilon_p = K_p V_{pmax}/(C_i+K_p)^2$ (Yin et al. 2016);
[c] these parameter values also given in Table 1 of the main text, based on *in vitro* measurements previously reported in the literature across various C_4 species.

Table S2 The 31-year mean (standard deviation in brackets) simulated aboveground mass at maturity of rice crop, for the present climate and the 2050 climate, either under potential or water-limited environments, in the site Shizukuishi. Simulation was conducted using two sets of C_4 kinetic parameter values given in Table S1, while other input-parameter values are as given in Table 1 for the C_4 type.

Route [a]	Production level	Climate type	Simulated aboveground mass (g m^{-2})	
			Yin et al. (2016) parameter	Boyd et al. (2015) parameter
4	Potential	Present	1944.3 (109.8)	1941.6 (108.9)
		2050	1952.5 (102.5)	1947.2 (102.7)
	Water-limited	Present	1423.1 (137.4)	1426.3 (140.9)
		2050	1404.6 (96.8)	1404.1 (99.3)
6	Potential	Present	2368.9 (108.5)	2370.5 (108.4)
		2050	2260.6 (106.1)	2238.4 (106.5)
	Water-limited	Present	1843.6 (155.8)	1842.5 (157.4)
		2050	1731.2 (119.9)	1730.8 (120.4)

[a] Route numbers as defined in Table 2 of the main text.

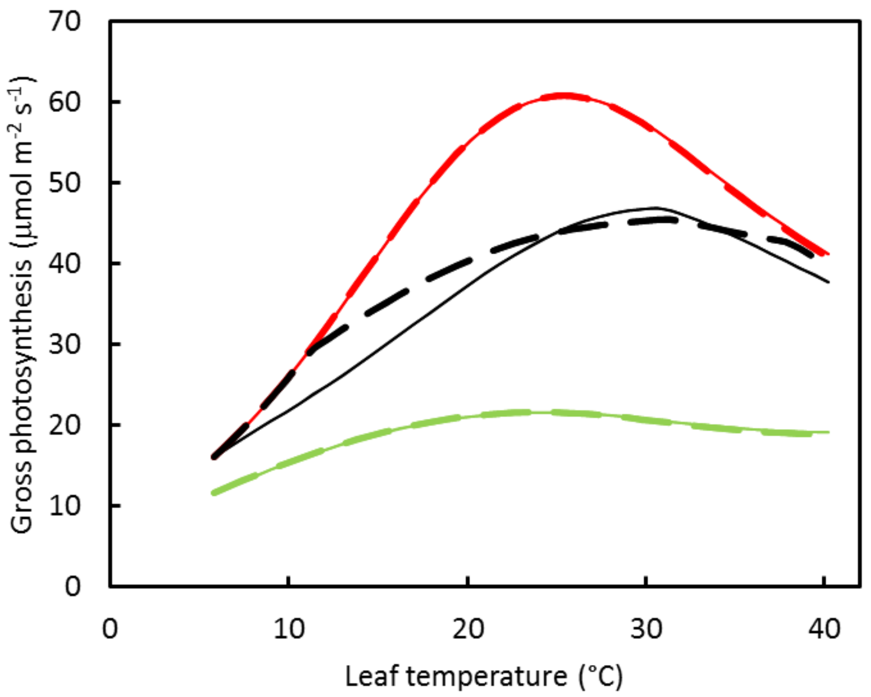

Fig. S3 Temperature response of gross C_4 leaf photosynthesis (A+R_d) modelled using parameter values of Yin et al. (1996) (continuous thin curves) or of Boyd et al. (2015) (thick dashed curves) under the conditions that ambient CO_2 level C_a = 400 μmol mol^{-1} and incident irradiance I_{inc} = 2000 μmol m^{-2} s^{-1} (red), or that C_a = 100 μmol mol^{-1} and I_{inc} = 2000 μmol m^{-2} s^{-1} (black), or that C_a = 400 μmol mol^{-1} and I_{inc} = 500 μmol m^{-2} s^{-1} (green). χ_{gbs25} = 0.007 (Table 1).

Supplementary Text 5
Sensitivity analysis with respect to ATP cost for cyanobacterial CCM

While it is believed that the ATP cost of bicarbonate transport required for cyanobacterial CCM may be lower than that of PEP regeneration required for the C_4 CCM (Price et al. 2013; Furbank et al. 2015), the exact ATP cost for cyanobacterial CCM is uncertain (Fridlyand et al. 1996; Price et al. 2011). In the simulation study described in the main text, we considered two well characterised, single-gene transporters (BicA and SbtA) based on the estimates by Price et al. (2011): the two transporters require 0.25 and 0.50 ATP per transport event, respectively. We assumed the simplest possible approach, total ATP requirement is the sum of the two; so, φ in eqn (5) is 0.75.

However, these transporters operate in parallel and adding their effects may not be accurate, because they have different kinetics (Price et al. 2011; McGrath & Long 2014) and thus their relative rates differ depending on bicarbonate concentration. The simplified model used in our simulation study does not allow the investigation of the effects of different kinetics of different transporters. The effect of the kinetics is presumably very important under low CO_2 conditions. For the relevant field environments, the effect of the kinetics cannot be straightforwardly implemented once modelling of g_s is also considered. For such conditions, we expect that the electron transport component of the model largely determines simulation results. As cyanobacterial CCM systems probably involve more than the two transporters (McGrath & Long 2014; Long et al. 2016), 0.75 might represent a lowest threshold of the ATP cost. Therefore, we conducted an analysis, by varying φ from 0.75 to 2.0 (the cost of C_4 CCM) at an interval of 0.25 (Table S3), to investigate how the simulated crop productivity of adding the complete cyanobacterial CCM system (Route 9) is affected by this uncertain parameter. The variation of input parameters x and f_{cyc} were adjusted accordingly (Table S3), following the method as described in the main text. We only use the potential production and the present climate scenario for this analysis; as in Supplementary Text 4, we again used 31-yr weather data of Shizukuishi for this illustration.

As expected, the 31-yr average of simulated aboveground crop mass decreased monotonically with increasing φ (Fig. S4). The simulated mass when φ was increased to 2.0 was ca 88% of that using the default φ value 0.75. This simulated productivity for Route 9 with $\varphi = 2.0$ was almost the same as that of Route 6 (i.e., the complete C_4 mechanism). Therefore, the simulated advantage of Route 9 over Route 6 shown in the main text was largely due to the presumed low ATP cost of cyanobacterial CCM. As the exact ATP cost for cyanobacterial CCM is unknown, our simulation results for cyanobacterial mechanisms (Routes 7-9) should be considered as tentative and be used mainly to support the conclusion, stated in the main text, that in addition to improving photosynthetic capacity and the effectiveness of CCM, improving quantum efficiency is also required for increasing canopy photosynthetic competence.

Table S3 Calculated values of parameters x and f_{cyc} in dependence of φ

φ	0.75	1.00	1.25	1.50	1.75	2.00
x	0.20	0.25	0.29	0.33	0.37	0.40
f_{cyc}	0.18	0.24	0.29	0.34	0.38	0.41

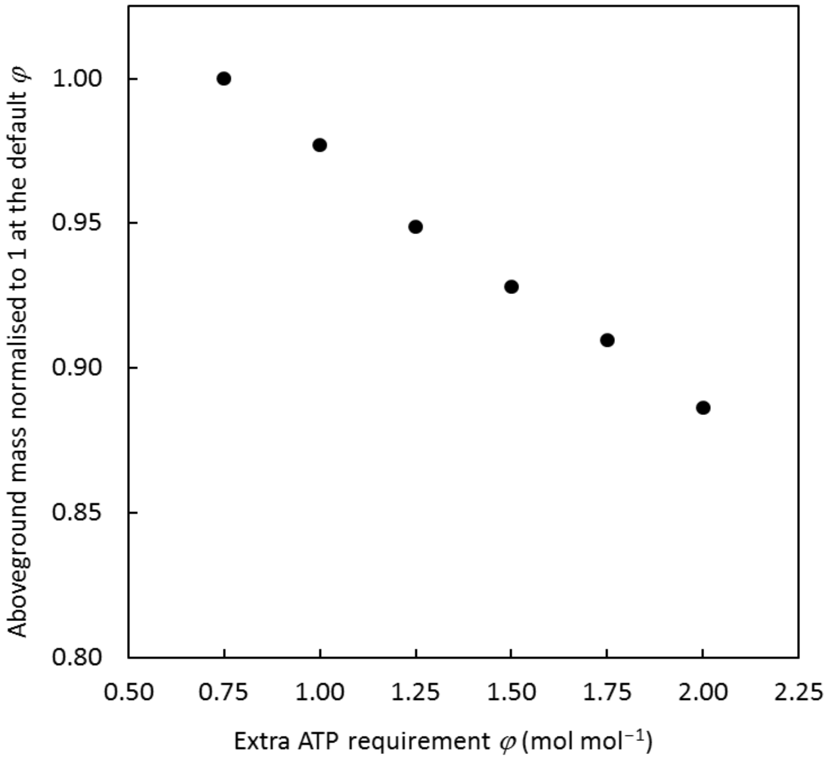

Figure S4 Simulated 31-yr average aboveground crop mass of Route 9 under different φ values assumed for extra ATP requirement per CO_2 assimilation, relative to that of the default extra ATP requirement (φ=0.75).

Supplementary Text 6
Description of the crop model GECROS (version 4.0)

GECROS (Genotype × Environment on CROp growth Simulator) is a generic crop model, which can be used for examining responses of crop yield, biomass and protein production in arable crops to both environmental variables and genotypic characteristics (for version 1.0, see Yin and van Laar 2005). The environmental variables as input to the crop model are radiation, temperature, wind speed, vapour pressure, and available amount of soil water and nitrogen.

Being arguably the most comprehensive crop model in which growth is assumed to be driven by photosynthesis, the model represents crop functions and interactive responses of contrasting growth components to environmental variables, thereby embodying physiological mechanisms that generate emergent feedback features. The contrasting components particularly emphasise carbon vs nitrogen interactions, root vs shoot, source activity vs sink capacity, and growth vs senescence relationships. The model uses innovative algorithms to summarise the current knowledge on individual physiological processes and their interactions and feedback mechanisms. It attempts to model crop growth process in a way that no area is treated in a trivial manner, unless unavoidable to the current understanding. GECROS also tries to maintain a balance between the robust model structure, the high computational efficiency, and the accurate model output. It requires little periodical destructive sampling to determine its input parameters.

Compared with many existing crop growth models, key features of GECROS are: (1) inherently coupled components for leaf nitrogen, conductance, photosynthesis, transpiration and leaf senescence; (2) carbon-nitrogen interaction to determine root-shoot partitioning, and sink demands to determine within-shoot partitioning; (3) seed protein production simulated in relation to crop nitrogen budget; (4) biosynthesis efficiency based on seed compositions; (5) applicable to most agricultural crops and to any production level (either potential, or water-limited, or nitrogen-limited) free of pest.

GECROS has since been updated a few times, particularly on calculating leaf photosynthesis rates. GECROS-v.1.0 calculates instantaneous leaf photosynthesis (A) from the C_3-photosynthesis model of Farquhar, von Caemmerer & Berry (1980; the FvCB model hereafter), assuming the C_i : C_a ratio only as a function of leaf-to-air vapour pressure difference (VPD). This approach was also applied to C_4 photosynthesis, with additional assumptions (i) to set internal [CO_2] to a high value, and (ii) to consider the extra ATP consumption by the C_4 CCM. These assumptions were abandoned in GECROS-v.2.0, which contains algorithms of Yin & Struik (2009) for coupled modelling of leaf photosynthesis and stomatal conductance g_s, while accounting for mesophyll conductance g_m for C_3 photosynthesis and bundle sheath conductance g_{bs} for C_4 photosynthesis. In GECROS-v.2.0, the analytical cubic polynomials (see Supplementary Text 1) simultaneously solve g_s, C_i, and leaf photosynthesis rate (A) for a given temperature. The effects of leaf N content on photosynthesis, g_s and transpiration are reflected by the effects of leaf N on model parameters like V_{cmax} and J_{max} (see Table 1 for parameter definitions). The GECROS model was later updated further (v.3.0; see Gu et al. 2014b) to allow an option that g_s and g_m may vary in parallel in response to environmental variables. For the present study, we used version 4.0, in which C_4 photosynthesis was particularly revised, based on recent estimation of model parameters (Yin et al. 2011; Yin & Struik 2012; Yin et al. 2016).

Detailed model algorithms for leaf photosynthesis in GECROS-v4.0 was given in the main text and Supplementary Texts 1 and 3. In this supplementary text, the coupling between leaf

photosynthesis and leaf transpiration, scaling to the canopy level, and how GECROS-v4.0 models other physiological processes are described.

Photosynthesis and transpiration

While photosynthesis and transpiration are different processes, they have to be coupled to enable a mechanistic way to quantify them, as described in the main text. The reason for the need of this coupling has two folds. First, responses of both processes to external conditions such as CO_2, vapour pressure and water availability are strongly regulated via stomata. Second, transpiration based on leaf energy balance determines the heat load and temperature of the leaf (T_l), and T_l affects the rates of most biochemical reactions of photosynthesis.

Potential leaf transpiration and its coupling with potential photosynthesis

When there is no water stress, photosynthesis rate largely determines the transpiration rate (Penning de Vries et al. 1989). The basic equation to estimate potential leaf transpiration, E_p, is the Penman-Monteith equation (Monteith 1973):

$$E_p = \frac{sR_n + \rho c_p D_a/(r_{bh} + r_t)}{\lambda\{s + \gamma[(r_{bw} + r_t + r_{sw,p})/(r_{bh} + r_t)]\}} \quad (1)$$

where R_n is net absorbed radiation, r_t is the turbulent resistance, r_{bh} and r_{bw} are the boundary layer resistance to heat and water transfer, respectively, $r_{sw,p}$ is the stomatal resistance to water transfer if there is no water stress, D_a is saturation vapour pressure deficit of the external air, ρc_p is volumetric heat capacity of air, λ is the latent heat of vapourisation of water, γ is the psychrometric constant. Eqn (1) somewhat differs from the original notation in that r_t is included in eqn (1) to allow for the case where movement of water and heat from within-canopy air spaces to the air above is important (Penning de Vries et al. 1989). Calculation of r_t, r_{bw}, r_{bh}, and R_n is given in Appendix A. The calculation of $r_{sw,p}$ is given in the main text.

The variable s in eqn (1) is given by:

$$s = [e_{s(T_l)} - e_{s(T_a)}]/(T_l - T_a) \quad (2)$$

where T_a is air temperature, $[e_{s(T_l)} - e_{s(T_a)}]$ is the difference in saturated water vapour pressure between leaf interior and external air. From the energy balance, leaf-to-air temperature differential, ΔT, has to be estimated by:

$$\Delta T = T_l - T_a = (r_{bh} + r_t)(R_n - \lambda E_p)/\rho c_p \quad (3)$$

There is a calculation loop in eqns (1), (2) and (3). A way to avoid this is to use an equation for saturated vapour pressure as a function of air temperature, $e_{s(T_a)}$ (Goudriaan and van Laar 1994):

$$e_{s(T_a)} = 0.611e^{17.4T_a/(239+T_a)} \quad (4)$$

The derivative of $e_{s(T_a)}$ with respect to T_a based on eqn (4) would give an estimate, which can be used as the proxy of s. However, an error is introduced by this approximation, especially when ΔT is high. McArthur (1990) has showed that this error can easily be eliminated by four to five iterations. In GECROS, one iteration procedure is performed, in which the derivative of eqn (4) is used for the first estimate of s as the input to eqn (2) that in turn gives input for calculating the second estimate of s according to eqns (2) and (3). This one-iteration procedure is considered sufficient to largely eliminate the error, since the first round estimate of ΔT is almost identical to its second round estimate in wide range of ΔT values (Yin and van Laar 2005). In this procedure,

T_a is used in leaf photosynthesis model to obtain the first round estimate of leaf photosynthesis. The first round estimate of ΔT is then used to model T_l, which is further used to advance for the second round estimate of P_p and E_p.

Actual leaf transpiration and photosynthesis if water stress occurs

When water supply from rooted soil layers does not meet requirement for potential transpiration, actual canopy transpiration is modelled to be the amount of water that is available in rooted layers for plant uptake. In such a case, actual transpiration determines actual photosynthesis (Penning de Vries et al. 1989). This methodology is supported by the fact that the effect of water stress on plants is largely mediated by stomata (Chaves 1991; Cornic 2000).

From eqn (1), one can derive an expression for the actual leaf stomatal resistance to water, $r_{sw,a}$ (Yin & van Laar 2015; also see eqn (18) in the main text). Since the actual stomatal resistance or conductance is now known, the actual leaf photosynthesis can be estimated as given in the main text. Again, an iteration approach involving eqns (2-3) is used to calculate the actual leaf temperature.

Spatial integration

In GECROS the concept of the sun-shade model (de Pury and Farquhar 1997) or the two-leaf model (Wang and Leuning 1998) is adopted, in which the canopy is divided into sunlit and shaded fractions and each fraction is modelled separately with a single-layer leaf model. Wang and Leuning (1998) indicated that the two-leaf model is computationally 10 times more efficient than the multi-layer model of Leuning et al. (1995) even though the latter model uses simple and fast numerical method, the Gaussian integration introduced by Goudriaan (1986) for crop modelling. De Pury and Farquhar (1997) showed that prediction of canopy photosynthesis by the two-leaf model is nearly the same as that given by a multi-layer model. The fraction of sunlit leaves at canopy depth L_i, $\phi_{su,I}$, is equal to the fraction of direct beam reaching that layer (Spitters 1986):

$$\phi_{su,i} = e^{-k_b L_i} \tag{5}$$

where k_b is beam radiation extinction coefficient of canopy. So, the sunlit fraction of the whole canopy, ϕ_{su}, is solved as:

$$\phi_{su} = \frac{1}{L}\int_0^L e^{-k_b L_i} dL_i = (1 - e^{-k_b L})/(k_b L) \tag{6}$$

and the fraction of shaded leaves of the canopy $\phi_{sh} = 1 - \phi_{su}$. Therefore, sunlit and shaded fractions of a canopy change during the day with solar elevation.

Radiation absorbed by a canopy, I_c, was determined as:

$$I_c = (1 - \rho_{cb})I_{b0}(1 - e^{-k_b' L}) + (1 - \rho_{cd})I_{d0}(1 - e^{-k_d' L}) \tag{7}$$

where I_{b0} and I_{d0} are incident direct-beam and diffuse radiation above the canopy, ρ_{cb} and ρ_{cd} are canopy reflection coefficient for direct-beam and diffuse light, respectively, k_b' and k_d' are extinction coefficients for beam and scattered beam, diffuse and scattered diffuse lights, respectively.

Radiation absorbed by the sunlit fraction of the canopy, $I_{c,su}$, is given as the sum of direct-beam, diffuse, and scattered beam components (de Pury and Farquhar 1997):

$$I_{\mathrm{c,su}} = (1-\sigma)I_{\mathrm{b0}}(1-e^{-k_{\mathrm{b}}L}) + (1-\rho_{\mathrm{cd}})I_{\mathrm{d0}}\frac{k_{\mathrm{d}}'[1-e^{-(k_{\mathrm{d}}'+k_{\mathrm{b}})L}]}{k_{\mathrm{d}}'+k_{\mathrm{b}}}$$
$$+ I_{\mathrm{b0}}\left\{(1-\rho_{\mathrm{cb}})\frac{k_{\mathrm{b}}'[1-e^{-(k_{\mathrm{b}}'+k_{\mathrm{b}})L}]}{k_{\mathrm{b}}'+k_{\mathrm{b}}} - (1-\sigma)\frac{1-e^{-2k_{\mathrm{b}}L}}{2}\right\} \quad (8)$$

where σ is leaf scattering coefficient.

Radiation absorbed by the shaded fraction of the canopy, $I_{\mathrm{c,sh}}$, can be calculated as the sum of incoming diffuse and scattered direct-beam radiation absorbed by these leaves. More simply, $I_{\mathrm{c,sh}}$ is calculated as the difference between the total radiation absorbed by the canopy, I_{c}, and the radiation absorbed by the sunlit fraction, $I_{\mathrm{c,su}}$ (de Pury and Farquhar 1997):

$$I_{\mathrm{c,sh}} = I_{\mathrm{c}} - I_{\mathrm{c,su}} \quad (9)$$

Eqns (7-9) were applied separately to visible or photosynthetically active radiation (PAR) and near-infrared radiation (NIR), because they have different values for σ, ρ_{cb}, ρ_{cd}, k_{b}, k_{b}' and k_{d}' (Goudriaan and van Laar 1994). Estimation of all extinction and reflection coefficients is given in Appendix B, and estimation of diffuse light fraction is given in Appendix C. The model assumes that half of the incident solar radiation is in the visible and other half is in the NIR waveband (Leuning et al. 1995).

The other important algorithms related to the spatial integration are for parameters that change with the depth of the canopy. These parameters are V_{cmax}, J_{max}, ε_{p}, g_{m} and g_{bs} for photosynthesis, and r_{bh} and r_{bw} for transpiration. These photosynthetic parameters are related to leaf nitrogen content n, r_{bh} and r_{bw} are related to wind speed u, and both n and u change with the depth of the canopy. To estimate these parameters for the entire canopy, and for the sunlit and shaded fractions of the canopy, photosynthetically active leaf nitrogen has to be scaled up. Assuming an exponential profile for the vertical decline of n in the canopy (Yin et al. 2000), photosynthetically active nitrogen for the entire canopy (N_{c}), for the sunlit fraction of the canopy ($N_{\mathrm{c,su}}$) and for the shaded fraction of the canopy ($N_{\mathrm{c,sh}}$), can be estimated by (Yin and van Laar 2005):

$$N_{\mathrm{c}} = n_0(1-e^{-k_{\mathrm{n}}L})/k_{\mathrm{n}} - n_{\mathrm{b}}L \quad (10)$$
$$N_{\mathrm{c,su}} = n_0[1-e^{-(k_{\mathrm{n}}+k_{\mathrm{b}})L}]/(k_{\mathrm{n}}+k_{\mathrm{b}}) - n_{\mathrm{b}}(1-e^{-k_{\mathrm{b}}L})/k_{\mathrm{b}} \quad (11)$$
$$N_{\mathrm{c,sh}} = N_{\mathrm{c}} - N_{\mathrm{c,su}} \quad (12)$$

where n_{b} is the base or minimum value of n, at or below which leaf photosynthesis is zero, n_0 is the nitrogen content for uppermost leaves, k_{n} is the leaf nitrogen extinction coefficient in the canopy (see later). For a given amount of canopy total nitrogen, n_0 can be estimated based on the exponential profile [see eqn (40) in a later section].

Similarly, assuming an exponential profile for the vertical decline of u in the canopy (Leuning et al. 1995), the boundary-layer conductance can be scaled up for the entire canopy (g_{bc}), for the sunlit fraction of the canopy ($g_{\mathrm{bc,su}}$), and for the shaded fraction of the canopy ($g_{\mathrm{bc,sh}}$), by (Yin and van Laar 2005):

$$g_{\mathrm{bc}} = 0.01\sqrt{u/w}(1-e^{-0.5k_{\mathrm{w}}L})/(0.5k_{\mathrm{w}}) \quad (13)$$
$$g_{\mathrm{bc,su}} = 0.01\sqrt{u/w}[1-e^{-(0.5k_{\mathrm{w}}+k_{\mathrm{b}})L}]/(0.5k_{\mathrm{w}}+k_{\mathrm{b}}) \quad (14)$$

$$g_{\mathrm{bc,sh}} = g_{\mathrm{bc}} - g_{\mathrm{bc,su}} \tag{15}$$

where k_{w} is the extinction coefficient of wind speed in the canopy, u is the wind speed at the top of the canopy (assuming equal to the speed observed meteorologically), and w is leaf width. The inverse of these estimates for the boundary-layer conductance gives the boundary-layer resistance for each fraction (sunlit, shaded) of the canopy. Eqns (13-15) are valid for estimating the boundary-layer conductance for heat. The boundary-layer conductance for water vapour has to be corrected by a factor of 1.075 = 1/0.93.

If water stress occurs, actual soil water available for crop uptake is partitioned between sunlit and shaded components according to their relative share of potential transpiration. The actual photosynthesis for each fraction is then estimated according to the method in the preceding section.

Temporal integration

For scaling up from instantaneous photosynthesis and transpiration to daily total, a numerical method, the Gaussian integration (Goudriaan 1986), is used here. The five-point method was adopted, using the normalised Gaussian distance $G_{\mathrm{x}}(i)$ = 0.04691, 0.23075, 0.50000, 0.76925 and 0.95309, with corresponding weights $G_{\mathrm{w}}(i)$ = 0.11846, 0.23931, 0.28444, 0.23931 and 0.11846 (Goudriaan and van Laar 1994). $G_{\mathrm{x}}(i)$ was used to select times during the day: $t(i) = 12 + 0.5D_{\mathrm{la}}G_{\mathrm{x}}(i)$, at which to evaluate the canopy variables (D_{la} is astronomic daylength; Appendix C). $G_{\mathrm{w}}(i)$ was applied to obtain daily integral: daily canopy photosynthesis (P_{C}) and daily canopy transpiration (E_{C}) are evaluated as:

$$P_{\mathrm{C}} = 3600 D_{\mathrm{la}} \sum_{i=1}^{5} P_{\mathrm{C}}(i) G_{\mathrm{w}}(i) \tag{16}$$

$$E_{\mathrm{C}} = 3600 D_{\mathrm{la}} \sum_{i=1}^{5} E_{\mathrm{C}}(i) G_{\mathrm{w}}(i) \tag{17}$$

where 3600 is seconds per hour, $P_{\mathrm{C}}(i)$ and $P_{\mathrm{C}}(i)$ are the instantaneous canopy photosynthesis and transpiration, respectively, at time $t(i)$, which are calculated from the approach outlined in the previous sections for spatial integration.

The temporal integration involves diurnal variation of environmental input variables. The daytime course of radiation and temperature is given in Appendix D. Instantaneous wind speed is assumed to be the same as the meteorologically recorded daily average, because diurnal course for wind speed is irregular, relative to that of temperature and radiation. The temporal course of daily amount of soil water available for daily transpiration could be simulated using a detailed process-based soil model. For model simplicity, it assumed to follow the course of radiation, though this assumption may not represent the reality.

Respiration

Cannell and Thornley (2000) proposed a framework that recognises individual relationships between respiration and each distinguishable biochemical process that it supports. The framework relates respiration to underlying biochemistry and physiology and provides opportunities to do this mechanistically and quantitatively, though many aspects of biochemistry underlying respiration remain uncertain. In this general framework, nine component processes

are distinguished: growth, symbiotic di-nitrogen (N_2) fixation, root nitrogen-uptake, nitrate reduction, other ion uptake, phloem loading, protein turnover, maintenance of cell ion concentrations and gradients, and any wasteful respiration. The first six of the nine processes can be quantifiable, whereas for the last three, together equivalent to the old classification of maintenance respiration (Penning de Vries et al. 1989), it is less easy to quantify them (Cannell and Thornley 2000) and an empirical approach will still be used.

Growth respiration

Here, 'growth' refers to only the process of biosynthesis within a growing organ and related phloem transport, excluding those processes such as mineral uptake and nitrogen reduction whose costs were included in the old classification of growth respiration (Penning de Vries et al. 1989). Growth respiration is defined by the concept 'growth yield', Y_G, the units of carbon (C) appearing in new biomass per unit glucose carbon utilised for growth.

The parameter Y_G can be calculated from the chemical composition of new plant material, based on the results of an exhaustive examination of the biochemical pathways for the production of protein, carbohydrate, lipid, lignin, and organic acid (Penning de Vries et al. 1974), by:

$$Y_G = \frac{30(0.444 f_{car} + 0.531 f_{pro} + 0.774 f_{lip} + 0.667 f_{lig} + 0.368 f_{oac})}{12(1.275 f_{car} + 1.887 f_{pro} + 3.189 f_{lip} + 2.231 f_{lig} + 0.954 f_{oac})} \quad (18)$$

where f_{car}, f_{pro}, f_{lip}, f_{lig}, f_{oac} are fraction of carbohydrates, proteins, lipid, lignin, and organic acid in new biomass material, 30 and 12 are the weight of per mol glucose (CH_2O) and carbon, coefficients in the numerator of eqn (18) are the fraction of carbon in carbohydrate, protein, lipid, lignin, and organic acid, respectively, and those in the denominator are the glucose requirement for producing per unit of these chemicals. These coefficients were taken from the summary analysis of Penning de Vries et al. (1989).

The composition of organs does not change significantly with environment. Some models do not consider differences in chemical composition between organs. In GECROS, it is assumed that it is the composition of the storage organ (seed hereafter) that varies among cultivars within a species whereas the compositional variation among vegetative organs is negligible. As a result, the value of Y_G for vegetative organ, $Y_{G,V}$, is given by a single value for a crop, whereas that for seed ($Y_{G,S}$) is allowed to vary, depending on its chemical composition. General information about chemical composition of organs can be found for main crops (Penning de Vries et al. 1989; Amthor 2000), if chemical analysis of specific crop organs is not performed.

Respiration for symbiotic N_2 fixation

Respiration supporting N_2 fixation occurs only in crops assimilating N_2 such as any leguminous component of the plant system. N_2 fixation requires both ATP and reductant. Nodule growth and maintenance and the concomitant respiration are also required for N_2 fixation. The estimated total cost for N_2 fixation is variable (depending on host species, bacterial strain, plant development) but lies mostly in the range 5-12 g C (g N fixed)$^{-1}$ (Cannell and Thornley 2000).

Respiration for ammonium nitrogen uptake

Basic data for estimating the cost of taking up ammonium-nitrogen are incomplete. Based on some assumptions, Cannell and Thornley (2000) have estimated that ammonium-nitrogen is

probably half as costly to take up as nitrate-nitrogen (see below). The respiratory cost for ammonium-nitrogen uptake is therefore set as 0.17 g C (g NH_4-N)$^{-1}$.

Respiration for nitrate uptake and reduction

The respiratory cost for nitrate-nitrogen uptake is 0.34 g C (g NO_3-N taken up)$^{-1}$ (Cannell and Thornley 2000). Unlike ammonium nitrogen that is, once taken up, available for plant metabolism without further respiratory costs, nitrate nitrogen must be reduced to the ammonium level. The full cost of nitrate reduction is 1.71 g C (g NO_3-N reduced)$^{-1}$ (Cannell and Thornley 2000). The total cost for both uptake and reduction of nitrate nitrogen is 2.05 g C (g NO_3-N)$^{-1}$.

Respiration for uptake of other ions

The uptake rate of minerals other than nitrogen is calculated from gross growth rate and ash content, which is assumed to be 0.05 g mineral per g dry matter (Thornley and Cannell 2000) though this value varies depending on crop and crop organs (Penning de Vries et al. 1989). The respiratory cost for ash uptake and within-plant transport has the value of 0.06 g C (g ash)$^{-1}$ (Thornley and Cannell 2000), comparable with the value of Penning de Vries et al. (1989), 0.12 g CH_2O per g minerals.

Respiration for phloem loading

Loading of sugars, amides and other substances into phloem for transport to sinks is an active process. Estimated costs for this loading and transport are included in eqn (18) by the glucose requirement for producing per unit of carbohydrates, proteins, lipid, lignin, and organic acid, assuming that loading requires 5.3% of the energy content of transported glucose (Penning de Vries et al. 1989).

Respiration for phloem loading here refers to (i) the cost specified by Thornley and Cannell (2000) for the transport of carbon from the shoot in the direction of the root, (ii) the cost of mobilising reserves in source organs. Both costs are assumed to have a value of 0.06 g C (g C loaded)$^{-1}$ (Amthor 2000; Thornley and Cannell 2000).

Other, less quantifiable respiration components

Cannell and Thornley (2000) refer processes such as protein turnover, maintaining cell ion gradients, futile cycles, and any use of the alternative pathway of respiration to as ‘residual maintenance respiration’. They indicated that evidence exists for a closer relation of residual maintenance respiration with tissue nitrogen content than with tissue mass. If related to mass, specific rate of maintenance respiration has been made of being organ-specific (Goudriaan and van Laar 1994) probably to account for the nitrogen concentration difference among the organs. Here, the ‘residual maintenance respiration’ (R_{rmr}) is related to the nitrogen content in the whole crop (N_T) as:

$$R_{rmr} = 44\varpi(N_T - n_{Lmin}W_S - n_{Rmin}W_R)/12 \quad (19)$$

where W_S and W_R are shoot and root weights, n_{Lmin} and n_{Rmin} are the minimum nitrogen concentration in leaf and root, respectively, which could be estimated from senesced plant materials, ϖ is daily specific rate of maintenance respiration, which is set in the model as 0.218 g C g^{-1} N d^{-1} (Ryan 1991). Eqn (19) can be multiplied by a term, e.g. using the concept of Q_{10}, to account for the effect of temperature on R_{rmr}. In GECROS, user can choose either include or not include this Q_{10} term, given the report that short-term respiratory response coefficients of plants

generally do not predict their long-term temperature response (Gifford 2003). For the simulation described in the main text, we included the term using $Q_{10} = 2$.

Nitrogen assimilation

The nitrogen uptake rate depends on both crop nitrogen demand and soil nitrogen availability. Soil may supply both ammonium and nitrate nitrogen. The availability of either ammonium or nitrate nitrogen from soil could be modelled by a process-based soil model. For crop simulation, supply of either ammonium or nitrate nitrogen should be considered as an input environmental variable to the crop model.

Nitrogen demand

The mechanism for crop nitrogen demand is poorly understood. It is assumed that crop demand has two components: deficiency-determined demand and growth activity-driven demand. The first component has commonly been used by modellers to define crop nitrogen demand (e.g. van Keulen and Seligman 1987). In the model, the second component plays a key role in defining crop nitrogen demand under most conditions; but the deficiency demand prevents extremely low nitrogen uptake by the crop especially when early growth is subjected to a severe stress.

The deficiency demand, N_{demD}, is the amount of nitrogen required to restore the actual nitrogen concentration (n_{act}) in the plant to the critical concentration, n_{cri} (Godwin and Jones 1991). The critical nitrogen curve of aboveground biomass over the growth cycle has often been shown (e.g. Justes et al. 1994). Therefore, N_{demD} is formulated on the basis of for shoot n_{cri} but correct for the difference of root and shoot contents as:

$$N_{\mathrm{demD}} = W_{\mathrm{S}}(n_{\mathrm{cri}} - n_{\mathrm{act}})(1 + N_{\mathrm{R}} / N_{\mathrm{S}}) / \Delta t \quad (20)$$

where W_{S} is crop aboveground dry weight, N_{S} and N_{R} are the amount of nitrogen in the shoot and the root, respectively, Δt is the time step of dynamic calculation in the model. The value of shoot n_{cri} is often defined as an empirical function of developmental stage (e.g. Robertson et al. 2002):

$$n_{\mathrm{cri}} = n_{\mathrm{cri0}} e^{-0.4\vartheta} \quad (21)$$

where ϑ is development stage, n_{cri0} is the critical aboveground nitrogen concentration at the onset of growth, which is crop or genotype-specific parameter.

The crop growth activity-determined nitrogen demand, N_{demA}, is calculated based on the assumption that crop takes up nitrogen in order to achieve the optimum nitrogen concentration (or in other term, optimum nitrogen/carbon ratio) that maximises its relative carbon gain. In analogy to the analysis of Hilbert (1990) for balanced growth conditions, achieving the optimum plant nitrogen/carbon ratio for a maximised relative carbon gain requires that relative root activity (σ_{N}) and relative shoot activity (σ_{C}) be balanced as (Yin and van Laar 2005):

$$\sigma_{\mathrm{N}} = \sigma_{\mathrm{C}}^2 / (\mathrm{d}\sigma_{\mathrm{C}} / \mathrm{d}\kappa) \quad (22)$$

where $\mathrm{d}\sigma_{\mathrm{C}}/\mathrm{d}\kappa$ is the first-order derivative of σ_{C} with respect to κ, the nitrogen/carbon ratio in the whole-plant. N_{demA} is then calculated by:

$$N_{\mathrm{demA}} = C_{\mathrm{R}} \sigma_{\mathrm{N}} = C_{\mathrm{R}} \sigma_{\mathrm{C}}^2 / (\mathrm{d}\sigma_{\mathrm{C}} / \mathrm{d}\kappa) \quad (23)$$

where C_{R} is the amount of carbon in roots. The value of σ_{C} is given by its definition as:

$$\sigma_{\mathrm{C}} = (\Delta C / \Delta t) / C_{\mathrm{S}} \quad (24)$$

where C_S is the amount of carbon in shoots; $\Delta C/\Delta t$ is daily crop carbon gain (often noted as NPP, net primary productivity), which is equal to $(12/44)Y_{G,V}(P_C-R_{ng})$, with R_{ng} being the sum of all non-growth components of respiration as discussed earlier. Here, $Y_{G,V}$ not $Y_{G,S}$ is used, because it is expected that plants take up most of the required nitrogen before the growth of the seeds (Sinclair and de Wit 1975).

Eqn (23), as well as carbon and nitrogen assimilate partitioning equations (see later), all involves the quantity $d\sigma_C/d\kappa$. Because $d\sigma_C/d\kappa$ cannot be calculated analytically given the sophistication of estimating σ_C in GECROS, it is numerically calculated by:

$$d\sigma_C / d\kappa = [\sigma_{C(\kappa+\Delta\kappa)} - \sigma_{C(\kappa)}] / \Delta\kappa \qquad (25)$$

where $\Delta\kappa$ is a small increment of κ; $\sigma_{C(\kappa)}$ and $\sigma_{C(\kappa+\Delta\kappa)}$ are relative shoot activities when plant nitrogen/carbon ratio is κ and $(\kappa+\Delta\kappa)$, respectively. Simulations showed that $d\sigma_C/d\kappa$ is not very sensitive to the value of $\Delta\kappa$, as long as $\Delta\kappa$ is sufficiently small. In GECROS, $\Delta\kappa$ was set to be 0.001κ.

Daily crop nitrogen demand, N_{dem}, is calculated as the maximum of N_{demD} and N_{demA}. However, the model does not allow N_{dem} to be more than the often observed upper threshold of daily nitrogen uptake N_{maxup} (Peng and Cassman 1998):

$$N_{dem} = \min[N_{maxup},\ \max(N_{demD}, N_{demA})] \qquad (26)$$

N_{maxup} is a model-input parameter, and is assumed to be crop or genotype specific.

Nitrogen fixation

In leguminous species, symbiotically fixed nitrogen is an important source of nitrogen absorbed by the crop. Though nitrogen is fixed in nodules, the model does not simulate the formation of nodules because of the lack of information. Instead, daily fixed nitrogen (N_{fix}) is determined simply as the minimum of two variables: the amount of demand for fixation (N_{fixD}) due to the shortfall of soil nitrogen supply in meeting crop nitrogen demand, and the amount allowed by plant energy supply (N_{fixE}). In the model, the shortfall of soil nitrogen supply of the preceding day is identified as N_{fixD}, reflecting the possibility that it may take some time for plants to act or signal for fixing required N. The variable of N_{fixE} is assumed simply because the nitrogen fixation is energy (ATP) demanding process (see the text on 'respiration'). The value of N_{fixE} is calculated by:

$$N_{fixE} = \max[0,\ (12/44)(P_C - R_{ngx})/c_{fix}] \qquad (27)$$

where R_{ngx} is the sum of all non-growth components of respiration but excluding the cost for the nitrogen fixation, c_{fix} is the carbon cost for fixing nitrogen, which, as said earlier, lies mostly in the range 5-12 g C (g N fixed)$^{-1}$ (Cannell and Thornley 2000).

The fixed nitrogen is assumed not to mix with soil nitrogen but to temporarily store in a reserve pool. The amount of fixed nitrogen in the pool, O_{Nfix}, is expressed as a state variable: its initial value is zero and its rate equation is given by:

$$dO_{Nfix} / dt = N_{fix} - \min(N_{dem}, O_{Nfix} / \tau_c) \qquad (28)$$

where τ_c is the time constant for utilisation of nitrogen in the pool (= 1 d).

The above equations are applied for leguminous crops. The value of N_{fix} in the above equations is switched to zero for non-leguminous crops.

Nitrogen uptake

Eqn (28) implies that plants take up nitrogen first from this pool and if insufficient, then from soil. The portion of N_{dem} not satisfied by $O_{\text{Nfix,}}$ $(N_{\text{dem}} - O_{\text{Nfix}} / \tau_{\text{c}})$, is replenished by any available soil nitrogen. Plants are assumed to take up soil ammonium and nitrate nitrogen impartially (Bradbury et al. 1993). Total daily nitrogen uptake by crop, N_{upt}, is then given by:

$$N_{\text{upt}} = N_{\text{uptA}} + N_{\text{uptN}} + \min(N_{\text{dem}}, O_{\text{Nfix}} / \tau_{\text{c}}) \quad (29)$$

where N_{uptA} and N_{uptN} are daily uptake of soil ammonium and nitrate nitrogen, respectively.

Assimilate partitioning and reserve dynamics

Partitioning of the newly produced carbon and absorbed nitrogen is modelled in two steps: first, between the root and the shoot, and then among organs within the shoot. Within-shoot organs include leaf, stem, and storage organ (seed, grain, or tuber; all referred to as seed hereafter). For root crops like potato, their storage organ is considered as a part of the shoot. Therefore, plant organs are defined in a functional rather than a morphological manner. For example, leaf is the photosynthetic organ and leaf area includes surface area in the stems or ears that also contribute to photosynthetic assimilate production (e.g. Biscoe et al. 1975).

Partitioning between root and shoot

The root-shoot partitioning is not assumed simply as a fixed tabular function of phenological development stage, as did in some existing models such as SUCROS (Goudriaan and van Laar 1994), because it is the root-shoot partitioning that might respond most to environmental changes (Wilson 1988). Instead, equations presented by Yin and Schapendonk (2004) are adapted here for the root-shoot partitioning of both carbon and nitrogen. The equations are based on the root-shoot functional balance theory (e.g. Charles and Edwards 1976; Brouwer 1983), with an incorporation of the mechanism that plants control root-shoot partitioning in order to maximise their relative carbon gain. The fraction of the newly assimilated carbon partitioned to the shoot ($\lambda_{\text{C,S}}$), and the fraction of newly absorbed nitrogen partitioned to the shoot ($\lambda_{\text{N,S}}$), are calculated by:

$$\lambda_{\text{C,S}} = \frac{1}{1 + (\eta / \sigma_{\text{C}}) \mathrm{d}\sigma_{\text{C}} / \mathrm{d}\kappa} \quad (30)$$

$$\lambda_{\text{N,S}} = \frac{1}{1 + [\eta N_{\text{R}} C_{\text{S}} / (\sigma_{\text{C}} N_{\text{S}} C_{\text{R}})] \mathrm{d}\sigma_{\text{C}} / \mathrm{d}\kappa} \quad (31)$$

where N_{S} and N_{R} are the amount of nitrogen in the shoot and the root, respectively; C_{S}, C_{R}, σ_{C}, and $\mathrm{d}\sigma_{\text{C}}/\mathrm{d}\kappa$ are the same as defined in an earlier section; and η is a variable, in analogy to the nitrogen/carbon ratio in newly formed biomass, as defined by:

$$\eta = \min(N_{\text{maxup}}, N_{\text{demA}}) / [(12/44) Y_{\text{G,V}} (P_{\text{C}} - R_{\text{ng}})] \quad (32)$$

Eqns (30) and (31) can be derived in analogy to the derivations as given by Yin and Schapendonk (2004). The model produces a pattern of root-shoot partitioning similar to the fixed pattern in most earlier Wageningen crop models using stage-dependent tabular functions, and can flexibly address the root-shoot ratios in response to environmental stresses such as water and nitrogen shortage (Yin and Schapendonk 2004).

Within-shoot carbon partitioning

Little is known about the mechanism that controls the within-shoot partitioning of carbon and nitrogen. It is assumed here that the strength of growing organs as sinks of available carbon determines the carbon partitioning. For such, a priority is specified: carbon goes first to the seed, secondly to the stem if the carbon source is more than the demand of seed as a sink, and then to leaf. Any further remainder carbon goes to shoot reserve pool. The dynamics of daily demand of either seed filling or stem growth for carbon is described by the differential form of a sigmoid function for asymmetric determinate growth (Yin et al. 2003a):

$$C_{\vartheta_i} = \omega_i C_{max} \frac{(2\vartheta_e - \vartheta_m)(\vartheta_e - \vartheta_i)}{\vartheta_e(\vartheta_e - \vartheta_m)^2} \left(\frac{\vartheta_i}{\vartheta_e}\right)^{\vartheta_m/(\vartheta_e - \vartheta_m)} \tag{33}$$

where $C_{\vartheta i}$ is the daily carbon demand at stage ϑ_i, ω_i is development rate at stage ϑ_i (see later), ϑ_e is the stage for end of growth (note that this equation assumes the start stage of growth is zero), ϑ_m is the stage at which the growth rate is maximum, C_{max} is the total demand of carbon by the end of growth. Instead of using classical growth equations such as the logistic equation, eqn (33) is chosen here because it ensures that the value of C_{max} is achieved at stage ϑ_e.

For seed filling, C_{max} is equal to $S_w S_f f_{c,S}/Y_{G,S}$ (where S_f = potential number of seeds, S_w = potential weight of a single seed, $f_{c,S}$ = fraction of carbon in seed biomass). S_w is a model-input parameter, and $f_{c,S}$ can be calculated according to the part in bracket in the numerator of eqn (18) (also see Penning de Vries et al. 1989). In the model, S_f is determined from the estimated amount of nitrogen and carbon accumulated before the end of seed-number determining period. The end of seed-number determining period (t_e) is a crop- or genotype-specific parameter, corresponding to the start of seed filling in determinate crops or a stage afterwards in indeterminate crops. For the stem growth, the value of C_{max} in eqn (33) is determined as $\rho H_{max} f_{c,V}/Y_{G,V}$ (where H_{max} = maximum plant height, $f_{c,V}$ = fraction of carbon in vegetative organ biomass, ρ is a constant as the slope of the linearity between stem biomass and plant height). If the currently assimilated available carbon does not satisfy the demand of a sink (seed or stem), the shortfall is added up to the demand of the following days till the shortfall is fulfilled by any new assimilated carbon. From this framework, the fraction of new shoot carbon partitioned to the seed ($\lambda_{C,seed}$), and to the stem ($\lambda_{C,stem}$) are determined. The fraction of new shoot carbon partitioned to the leaf ($\lambda_{C,leaf}$) is determined, depending on whether it is carbon or nitrogen that limits the growth of canopy:

$$\lambda_{C,leaf} = \begin{cases} 0 & L_C \geq L_N \\ 1 - \lambda_{C,seed} - \lambda_{C,stem} & L_C < L_N \end{cases} \tag{34}$$

where L_C and L_N are carbon-limited and nitrogen-limited leaf area index, respectively (see later). Both $\lambda_{C,stem}$ and $\lambda_{C,leaf}$ are, however, fixed to zero after the end of seed-number determining period, after which no vegetative growth is expected. The fraction of carbon partitioned to leaf, predicted by eqn (34), can fluctuate for some days in the middle growth phase when L_C and L_N become limiting in tandem.

Carbon reserves and their remobilization

The fraction of new shoot carbon going to the shoot reserve pool ($\lambda_{C,Sres}$) is then calculated by:

$$\lambda_{C,Sres} = 1 - \lambda_{C,seed} - \lambda_{C,stem} - \lambda_{C,leaf} \tag{35}$$

GECROS also assumes existence of the root carbon reserve pool, to which new root carbon goes. The fraction of new root carbon going to root reserve pool is either 0 or 1, depending on whether

it is carbon or nitrogen that limits the growth of structural root biomass. Nitrogen-determined root mass is given in the next section [see eqn (44)].

If the new shoot carbon is less than the demand for carbon by seed filling, the carbon in reserve pools, if any, is remobilized to fill the shortfall. It is assumed that shoot and root carbon reserves are remobilized impartially. Remobilization is an active process that needs energy to support; the costs is assumed to be 0.06 g C (g C remobilized)$^{-1}$ (Thornley and Cannell 2000). Upon conversion of reserves to structural seed biomass, there are carbon losses due to growth respiration. The losses of carbon due to both the growth respiration and the cost of remobilization contribute to the slight reduction of total crop weight, as frequently observed when crop approaches to maturity. When the amount of both new shoot carbon and remobilized carbon does not suffice the requirement for seed growth, the weight of seeds that are expected to fill is subject to decline.

Within-shoot nitrogen partitioning

For the intra-shoot nitrogen partitioning, certain seed and stem nitrogen concentrations are assumed. The nitrogen requirements for seed growth in various crops (Sinclair and de Wit 1975) are used as basic data to quantify seed nitrogen concentration under standard conditions (n_{SO}). Because organs are defined functionally, the photosynthetic part of stems is considered as ‘leaves’. Based on this framework of functional organs, constant minimum structural-stem nitrogen concentration (n_{Smin}) and constant reserve nitrogen concentration (n_{RV}) are assumed for each crop. If the requirement for nitrogen by seeds, stems and reserves is met, the leftover of new shoot nitrogen goes to functional ‘leaves’. Otherwise, nitrogen in leaves and roots will be remobilized impartially. The remobilization of nitrogen from leaves and roots reduces leaf and root nitrogen and thereof facilitates leaf and root senescence, reflecting the phenomenon of so-called ‘*self-destruction*’ termed by Sinclair and de Wit (1975). The total nitrogen in leaves and in roots available for remobilization is estimated by $(N_{\mathrm{LV}} - n_{\mathrm{Lmin}} W_{\mathrm{LV}})$ and $(N_{\mathrm{R}} - n_{\mathrm{Rmin}} W_{\mathrm{R}})$, respectively. When the amount of both new shoot nitrogen and remobilized nitrogen still does not suffice the requirement for nitrogen by seed growth, the nitrogen concentration of newly formed seed biomass, and of total seed biomass thereof, is subject to decline till a pre-defined minimum seed nitrogen concentration, which is a fraction (q) of n_{SO}. If this minimum seed nitrogen is reached, the filling of carbon to seeds stops. Seed protein quantity is estimated from the total accumulated nitrogen in seeds multiplied by a standard conversion factor 6.25.

Crop morphology, senescence, and crop phenology

Leaf area index

The green leaf area index (L) is modelled, according to the guidelines given by Yin et al. (2000, 2003b), as the minimum of L_{C} and L_{N}. The value of L_{N} is calculated by the equation presented by Yin et al. (2000):

$$L_{\mathrm{N}} = (1/k_{\mathrm{n}})\ln(1 + k_{\mathrm{n}} N_{\mathrm{LV}} / n_{\mathrm{b}}) \qquad (36)$$

where N_{LV} is the amount of nitrogen in leave of the canopy, k_{n} is the extinction coefficient of leaf nitrogen, and n_{b} is the base value of leaf nitrogen at or below which there is no active photosynthesis.

The value of L_{C} is assumed as a state variable, whose rate of increment, ΔL_{C}, is estimated in two phases (Yin et al. 2003b):

$$\Delta L_{\mathrm{C}} = \begin{cases} (n_{\mathrm{bot}}\Delta N_{\mathrm{LV}} - N_{\mathrm{LV}}\Delta n_{\mathrm{bot}})/[n_{\mathrm{bot}}(n_{\mathrm{bot}} + k_{\mathrm{n}} N_{\mathrm{LV}})] & L_{\mathrm{C}} \le 1 \\ s_{\mathrm{la}}\Delta C_{\mathrm{LV}} / f_{\mathrm{c,V}} & L_{\mathrm{C}} > 1 \end{cases} \tag{37}$$

where s_{la} is specific leaf area constant, ΔC_{LV} is the increment of carbon in leaves, ΔN_{LV} is the increment of nitrogen in leaves, n_{bot} is the nitrogen content of the bottom leaves in canopy. In the model, n_{bot} is described as an additional state variable, whose rate of increment, Δn_{bot}, is calculated by:

$$\Delta n_{\mathrm{bot}} = (n_{\mathrm{botE}} - n_{\mathrm{bot}}) / \Delta t \tag{38}$$

where n_{botE} stands for the value of n_{bot} expected from the exponential profile of leaf nitrogen content. The rationale of using eqns (37) and (38) was outlined by Yin et al. (2003b).

For the given value of N_{LV}, the nitrogen content of bottom leaves in the canopy is expected from the exponential profile as:

$$n_{\mathrm{botE}} = k_{\mathrm{n}} N_{\mathrm{LV}} e^{-k_{\mathrm{n}}L} /(1 - e^{-k_{\mathrm{n}}L}) \tag{39}$$

Similarly, based on the exponential profile, the nitrogen content of top leaves in the canopy, n_0 [required by eqns (10-11)], is estimated by:

$$n_0 = k_{\mathrm{n}} N_{\mathrm{LV}} /(1 - e^{-k_{\mathrm{n}}L}) \tag{40}$$

These equations use a critical parameter k_{n}, which is also required by eqns (10-12). Since k_{n} and its temporal variation are not amenable to experimental collection, the model estimates k_{n} by (Yin et al. 2003b):

$$k_{\mathrm{n}} = \frac{1}{L_{\mathrm{T}}} \ln\left[\frac{k_{\mathrm{r}}(N_{\mathrm{LV}} - n_{\mathrm{b}}L_{\mathrm{T}}) + n_{\mathrm{b}}(1 - e^{-k_{\mathrm{r}}L_{\mathrm{T}}})}{k_{\mathrm{r}}(N_{\mathrm{LV}} - n_{\mathrm{b}}L_{\mathrm{T}})e^{-k_{\mathrm{r}}L_{\mathrm{T}}} + n_{\mathrm{b}}(1 - e^{-k_{\mathrm{r}}L_{\mathrm{T}}})} \right] \tag{41}$$

where L_{T} is total (green + senesced) leaf area index; k_{r} is the extinction coefficient for PAR, and is set here to equal $k_{\mathrm{d}}^{'}$ for PAR [see eqn (B4)], assuming diffuse component of PAR is dominant for determining nitrogen distribution in a canopy (Anten 1997). While eqn (41) shows that there are four variables (k_{r}, N_{LV}, n_{b}, and L_{T}) that affect k_{n}, it is the value of k_{r} that almost uniquely determines k_{n}, reflecting leaf nitrogen acclimation controlled by local light environment in crop canopy.

Leaf and root senescence

As can be seen above, GECROS predicts leaf area index as the interactive result of leaf carbon and nitrogen dynamics. One of the advantages of this approach is to enable leaf senescence to be modelled in a simple but robust way (Yin et al. 2000). The loss rate of leaf biomass due to senescence at a time step ($\Delta W_{\mathrm{LV}}^{-}$) can be estimated simply by:

$$\Delta W_{\mathrm{LV}}^{-} = [L_{\mathrm{C}} - \min(L_{\mathrm{C}}, L_{\mathrm{N}})]/(s_{\mathrm{la}}TC_{\mathrm{S}}) \tag{42}$$

where TC_{S} is time constant for senescence. The numerator of eqn (42) gives estimate of the leaf area senesced at a time step. Eqn (42) predicts increased leaf senescence during seed filling because L_{N} becomes increasingly small due to the withdrawal of leaf nitrogen to seeds, thereby reflecting well the '*self-destruction*' phenomenon defined by Sinclair and de Wit (1975). The loss rate of leaf nitrogen, i.e. the amount of nitrogen going to the senesced leaf materials ($\Delta N_{\mathrm{LV}}^{-}$), can be estimated as $n_{\mathrm{Lmin}}\Delta W_{\mathrm{LV}}^{-}$.

Under the conditions when seed filling is retarded due to environmental stresses such as water shortage, eqn (42) can result in no senescence because limited seed filling requires little

remobilization of leaf nitrogen. The senescence rate in such cases, i.e. if eqn (42) predicts no senescence after active seed filling is initiated, is described as $r_{LV}W_{LV}$, where r_{LV} refers to relative leaf death rate as defined by Goudriaan and van Laar (1994). The loss rate of leaf nitrogen with the senesced leaf materials in such conditions is estimated as ΔW_{LV}^{-} multiplied by leaf nitrogen concentration (n_L). The use of n_L rather than n_{Lmin} is to mimic the increased leaf nitrogen volatilisation under stress conditions such as drought (Weiland et al. 1982).

In analogy to the modelling of leaf senescence, the loss rate of structural root biomass due to senescence at a time step (ΔW_R^{-}) is estimated by:

$$\Delta W_R^{-} = [W_{SR} - \min(W_{SR}, W_{SR,N})] / TC_S \tag{43}$$

where $W_{SR,N}$ is nitrogen-determined W_{SR}, which is estimated by an equation similar to eqn (36):

$$W_{SR,N} = (1/k_{Rn}) \ln(1 + k_{Rn} N_{SR} / n_{Rmin}) \tag{44}$$

where N_{SR} is structural root nitrogen, k_{Rn} is extinction coefficient of root nitrogen concentration. The value of N_{SR} can be calculated as the difference between total root nitrogen (N_R) and root reserve pool nitrogen, assuming that nitrogen concentration in root reserve pool is n_{Rmin}. The loss rate of root nitrogen, for the amount of nitrogen going to the senesced root materials (ΔN_R^{-}), can then be estimated as $n_{Rmin}\Delta W_R^{-}$.

Plant height and root depth

Estimation of plant height is required for calculating carbon demand by stem growth. Plant height is quantified as a state variable, and its rate is estimated by the differential form of a sigmoid growth function with the same form as eqn (33), assuming that maximum plant height, H_{max}, is reached at the onset of seed filling for determinate crops or the halfway between the onset of seed filling and t_e for indeterminate crops. Effects of any abiotic stress on plant height are accounted for by multiplying the rate equation with a factor determined as the ratio between carbon assimilates available for stem growth at current time step and carbon demands for stem growth in the preceding time step. The value of this factor is limited within the range between 0 and 1. Use of the C demand in the preceding time step avoids calculation loop.

The rooting depth, D, is an important element to predict because only water and nutrients in the rooted soil layer are subject to plant uptake. In the model, D is described in the same way as for aboveground plant height, subject to a crop- or genotype- specific maximum rooting depth (D_{max}).

Phenological development

Phenology provides the temporal framework for simulating a number of processes. An index variable, development stage (ϑ), is defined as a state variable, having a dimensionless value of 0 at seedling emergence, 1 at the start of seed filling, and 2 at seed maturity. Development stage is the accumulation of daily development rate (ω_i), which has a unit of d^{-1}. The value of ω is calculated separately for pre-seed filling (vegetative growth) period and for the seed-filling (reproductive growth) period. For the vegetative period, ω is calculated by:

$$\omega_i = \begin{cases} g(T)/m_V & \vartheta \le \vartheta_1 \quad \text{or} \quad \vartheta \ge \vartheta_2 \\ g(T)h(D_{lp})/m_V & \vartheta_1 < \vartheta < \vartheta_2 \end{cases} \tag{45}$$

where m_V is the minimum number of days for the vegetative period when the photothermal environment is at the optimum, ϑ_1 and ϑ_2 are the development stage for the start and the end of photoperiod-sensitive phase, respectively, D_{lp} is photoperiodic daylength [see eqn (C7)]. The temperature effect function, $g(T)$, is defined, using the flexible bell-shaped nonlinear function (Yin et al. 1995) as

$$g(T) = \left[\left(\frac{T_c - T}{T_c - T_o} \right) \left(\frac{T - T_b}{T_o - T_b} \right)^{\frac{T_o - T_b}{T_c - T_o}} \right]^{c_t} \tag{46}$$

where c_t is temperature response curvature coefficient, T_b, T_o, and T_c are the base, the optimum, and the ceiling temperature for phenological development [i.e. $g(T) = 0$ if $T \leq T_b$ or $\geq T_c$].

Because temperature is diurnally fluctuating under field conditions, $g(T)$ is estimated on an hourly basis and hourly $g(T)$ values are averaged for the daily value. The hourly temperature is estimated from daily maximum and minimum temperature by a sine function assuming the daily maximum occurs at 14:00 each day [see eqn (D3)]. Crop difference in phenological response to temperature lies not in the value of c_t but in the cardinal temperature (T_b, T_o, and T_c). In case of the lack of data for determining c_t by curve fitting, c_t can be assumed to be equal to 1.

Eqn (45) indicates that daily development rate during the vegetative growth period is modified by photoperiod, if cultivar or genotype is photoperiod-sensitive. This modification is reflected by $h(D_{lp})$, which has a value between 0 and 1 with:

$$h(D_{lp}) = 1 - p_{sen}(D_{lp} - M_{op}) \tag{47}$$

where M_{op} is the maximum optimum photoperiod for a short-day crop (about 11 h) or the minimum optimum photoperiod for a long-day crop (about 18 h); p_{sen} is the photoperiod-sensitivity parameter, being positive for short-day crops, negative for long-day crops. A zero value of p_{sen} characterises absolutely insensitive cultivars of any crop. Genotypic difference within a crop in phenological response to photoperiod is assumed not in M_{op} but mainly in p_{sen}, ϑ_1 and ϑ_2.

Development rate for seed filling phase is calculated dependent on temperature only, using genotypic coefficient m_R, the minimum number of days for the seed-filling period when the temperature is at its optimum. The effect of temperature on ω of this period is also based on eqn (46), but with a restriction that T is set to T_o if $T > T_o$ to abolish the decline of development rate at high temperatures. This restriction is incorporated to account for the accelerated seed filling when plants are exposed to high temperatures.

Input requirements and model implementation in FST

The inter-connection of the model components as outlined above is schematically shown in the relational diagram of GECROS (Figure S5). The main program of the model runs on a daily time step (i.e. time step Δt = 1 d), whereas some physiological processes (e.g. phenology, photosynthesis and transpiration, and maintenance respiration in response to temperature) are simulated in their subroutines in shorter time steps.

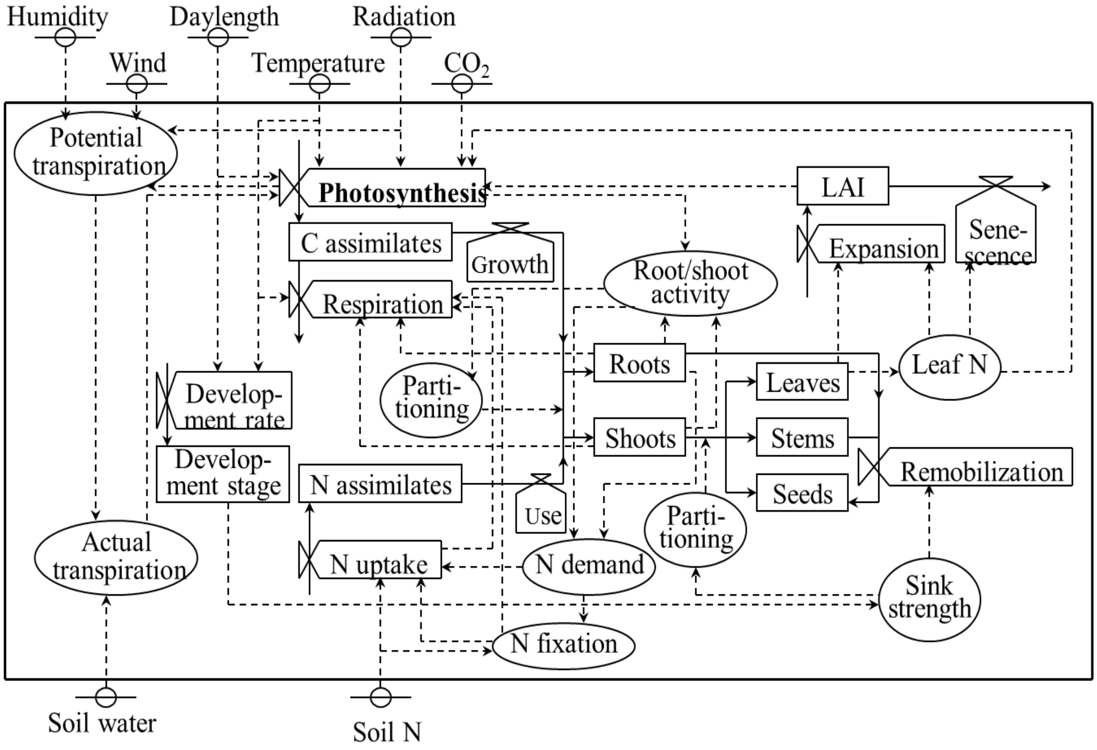

Fig. S5. The relational diagram, drawn using symbols of Forrester (1961), for the crop model GECROS (after Yin and van Laar 2005).

Initialisation, and biophysical inputs

Initial values of state variables have to be provided. The default initial condition is set at crop seedling emergence. Initial values of W_S and W_R at seedling emergence could be determined from seeding rate and seed weight, assuming crop-specific germination efficiency, ε_g (Penning de Vries et al. 1989) and a standard initial shoot ratio (υ_{C0}). But we advise that model users set the initial values of W_S and W_R for their particular situations. Initial N_S and N_R can be set, using n_{cri0} as shoot nitrogen concentration at emergence and a certain initial shoot nitrogen ratio (υ_{N0}).

Required daily weather inputs are: global radiation, minimum air temperature, maximum air temperature, vapour pressure, wind speed, and precipitation. Latitude of the location should also be provided to calculate daylength.

The other required model inputs are daily supply of water and nitrogen available for crop uptake. These two input variables can be provided by soil model simulations, once the GECROS crop model is coupled with a process-based soil model (see Yin and van Laar 2005). The coupled model can then be used for examining crop production in response to not only physical environmental conditions but also edaphic variables and managerial options (amount and timing of irrigation, amount and timing of nitrogen fertilization). The other managerial factor – to which the model responds – is rate and timing of sowing.

Model constants and parameters

A few model constants are basically related to physical relations for transpiration (ρc_p, λ, γ, and B_Z). Those biochemical coefficients for leaf photosynthesis are given in Table 1 of the main text.

Those parameters that vary either with crops or with genotypes within a crop are given in the original version of GECROS (Yin and van Laar 2005), and some of them are specified in Table 3 of the main text for the present simulation.

Model implementation in FST

GECROS is currently implemented in the FST (FORTRAN Simulation Translator) computer language (van Kraalingen et al. 2003). The FST language and the corresponding FST software feature a powerful and easy-to-use simulation language providing clear error message (van Kraalingen et al. 2003). The mathematical meaning of some intrinsic FST functions, which are not provided by standard FORTRAN, were frequently used in the GECROS source codes.

The most important programming guideline for a model to be coded in the FST is that it uses the state-rate concept of simulation, known as the state variable approach (Penning de Vries et al. 1989). The state variables are described in FST by the INTGRL function. Usually, the largest part of source codes of a FST program is to deal with algorithms for calculating rate variables and their associated auxiliary variables. The new FST version (4.12) is available for downloading at http://models.pps.wur.nl/node/970. This FST version works with freely available Fortran (GFortran).

Finally it is worthy to note that GECROS is a crop model. For model applications under field conditions, GECROS needs to be linked with a process-based soil model. The points for coupling between GECROS and soil models then need to be identified. Soil model predicts the amount of water and mineral nitrogen available in rooted soil layer for crop absorption. The crop model predicts the amount of organic carbon and nitrogen from senesced leaf and root materials entering to the soil as litters. An example model for simulating soil processes is described by Yin and van Laar (2005). Users may link GECROS to their own soil models in order to cope with model applications at the field level.

Appendices for Supplementary Text 6

Appendix A *Calculation of input variables of eqn* (1): r_t, r_{bh}, r_{bw}, and R_n

The turbulent resistance, r_t, which has the same value for heat, CO_2 and water transfer (Goudriaan 1982), is calculated by (Goudriaan et al. 1984):

$$r_t = 0.74\{\ln[(2-0.7H)/(0.1H)]\}^2/(0.16u) \quad \text{(A1)}$$

where u is the wind speed above the canopy, H is crop height.

The leaf boundary layer resistance to heat, r_{bh}, is estimated as:

$$r_{bh} = 100\sqrt{w/u} \quad \text{(A2)}$$

where w is leaf width. The leaf boundary layer resistance to water vapour, r_{bw}, is estimated by:

$$r_{bw} = 0.93 r_{bh} \quad \text{(A3)}$$

where the factor 0.93 allows for the difference in velocity of boundary-layer transfer between heat and water vapour (Goudriaan and van Laar 1994).

Net absorbed radiation by leaves, R_n, is the difference of the absorbed short-wave radiation (PAR plus NIR) and the outgoing long-wave radiation ($R^{\uparrow}$). The absorbed total short-wave radiation is given as the sum of absorbed PAR and absorbed NIR, according to eqn (7).

Following the algorithms described by van Laar et al. (1997), $R^{\uparrow}$ is approximated by three semi-empirical functions, accounting for temperature, vapour pressure in the atmosphere and sky clearness:

$$R^{\uparrow} = B_z (T_1 + 273)^4 f_{vap} f_{clear} \phi_i \tag{A4}$$

$$f_{vap} = 0.56 - 0.079\sqrt{10V} \tag{A5}$$

$$f_{clear} = 0.1 + 0.9 \max\{0, \min[1, (\tau - 0.2)/0.5]\} \tag{A6}$$

where B_z is Stephen-Boltzmann constant, ϕ_i is the fraction of a leaf class (= ϕ_{su} and ϕ_{sh} for sunlit and shaded leaves, respectively), V is vapour pressure, τ is atmospheric transmissivity (see Appendix C), 0.2 and 0.5 are empirical constants (0.2 is the atmospheric transmissivity under an overcast sky, and 0.5 is the additional transmissivity from direct radiation.

Appendix B *Canopy extinction and reflection coefficients*

Extinction coefficient for beam radiation required for eqn (5) for a canopy is given by (Goudriaan 1988):

$$k_b = O_{av} / \sin\beta \tag{B1}$$

where $\sin\beta$ is given by eqn (C3) in Appendix C, O_{av} is the average projection of leaves in the direction of a solar beam. Assuming that the leaves in a canopy have a uniform azimuth orientation, O_{av} can be found as:

$$O_{av} = \begin{cases} \sin\beta \sin\beta_L & \beta \geq \beta_L \\ 2[\sin\beta \cos\beta_L \arcsin(\tan\beta / \tan\beta_L) + \sqrt{\sin^2\beta_L - \sin^2\beta}]/\pi & \beta < \beta_L \end{cases} \tag{B2}$$

where β_L is the leaf inclination angle in a canopy, being crop or cultivar-specific input parameter.

Overall extinction coefficient for beam and scattered beam radiation, $k_b^{'}$, required for eqns (7) and (8) is given by (Goudriaan and van Laar 1994):

$$k_b^{'} = k_b \sqrt{1-\sigma} \tag{B3}$$

where σ is the scattering coefficient for leaves (typically 0.2 for PAR and 0.8 for NIR).

Overall extinction coefficient for diffuse and scattered diffuse radiation, $k_d^{'}$, can be derived by taking the profile of diffuse radiation in the canopy to be a summation of profiles each originating from a separate ring zone of the sky. Goudriaan (1988) showed that the effects of leaf angle distribution can be accurately described by using as few as three 30° zone classes (0-30, 30-60, 60-90°). The extinction coefficient for each zone can be found by substituting β in eqns (B1-B2) by the elevation of the centre of each zone class (i.e. 15, 45 and 75°); and these coefficients are termed here as k_{b15}, k_{b45} and k_{b75}, respectively. The value for $k_d^{'}$ can then be calculated for standard overcast sky conditions as (Goudriaan 1988):

$$k_d^{'} = -(1/L_T)\ln\left(0.178 e^{-k_{b15}\sqrt{1-\sigma}L_T} + 0.514 e^{-k_{b45}\sqrt{1-\sigma}L_T} + 0.308 e^{-k_{b75}\sqrt{1-\sigma}L_T}\right) \tag{B4}$$

where L_T is total leaf area index; the weights 0.178, 0.514 and 0.308 represent the contributions from the three zones of a standard overcast sky.

Canopy reflection coefficient for beam radiation, ρ_{cb} in eqns (7)-(8), is related to the canopy reflection coefficient for horizontal leaves, ρ_h, by (Goudriaan 1977):

$$\rho_{cb} = 1 - e^{-2\rho_h k_b/(1+k_b)} \tag{B5}$$

where

$$\rho_h = (1 - \sqrt{1-\sigma})/(1 + \sqrt{1-\sigma}) \tag{B6}$$

Canopy reflection coefficient for diffuse radiation, ρ_{cd}, could be calculated by numerical integration of ρ_{cb} and the sky radiance over the hemisphere of the sky (De Pury and Farquhar 1997). In GECROS, ρ_{cd} is estimated separately for PAR and for NIR, each as a weighted average of values from eqn (B5) using the elevation of three zone classes (15, 45 and 75°) across the sky.

Appendix C *Solar elevation, daylength, and direct and diffuse solar radiation*

According to Goudriaan and van Laar (1994), the direct radiation incident on a horizontal plane at the Earth's surface, S_o, may be written as

$$S_o = \tau S_c \sin\beta \qquad \text{(C1)}$$

where τ is the atmospheric transmissivity, S_c is solar constant, referring to the radiation normal to the sun's beam outside the Earth's atmosphere:

$$S_c = 1367[1 + 0.033\cos(2\pi(t_d - 10)/365)] \qquad \text{(C2)}$$

where t_d is the day of the year (1 = January 1). The solar elevation $\sin\beta$ is:

$$\sin\beta = a + b\cos[2\pi(t_h + 12)/24] \qquad \text{(C3)}$$

where t_h is the time of a day, and

$$a = \sin(\pi\zeta/180)\sin\delta, \qquad b = \cos(\pi\zeta/180)\cos\delta \qquad \text{(C4)}$$

with

$$\delta = -\arcsin\{\sin(23.45\pi/180)\cos[2\pi(t_d + 10)/365]\} \qquad \text{(C5)}$$

where ζ is the latitude, and δ is the declination of the sun with respect to the equator.

The quantities a and b may be used to evaluate the astronomic daylength, D_{la}, and the photoperiodic daylength, D_{lp}, in hours:

$$D_{la} = 12[1 + (2/\pi)\arcsin(a/b)] \qquad \text{(C6)}$$

$$D_{lp} = 12\{1 + (2/\pi)\arcsin[(-\sin(\alpha\pi/180) + a)/b]\} \qquad \text{(C7)}$$

where α is the sun angle below horizon, for including civil twilight as photoperiodic daylength. The expression for D_{la} is required for the Gaussian integration [see eqns (16-17)] and for calculating daytime course of temperature [eqn (D2)], and the expression for D_{lp} is required for estimating photoperiodic response of phenological development [see eqn (47)].

The quantities a, b and D_{la} may also be used to calculate integral of $\sin\beta$ over the day, $D_{\sin\beta}$:

$$D_{\sin\beta} = 3600[D_{la}a + (24/\pi)b\sqrt{1-(a/b)^2}] \qquad \text{(C8)}$$

Eqn (C8) could be used to obtain the daily extraterrestrial radiation. However, a quantity equivalent to $D_{\sin\beta}$, but with a correction for lower atmospheric transmission at lower solar elevations, may be used (Goudriaan and van Laar 1994). This quantity is noted as $D_{\sin\beta e}$:

$$D_{\sin\beta e} = 3600\{D_{la}[a + 0.4(a^2 + 0.5b^2)] + (12/\pi)b(2 + 1.2a)\sqrt{1-(a/b)^2}\} \qquad \text{(C9)}$$

$D_{\sin\beta e}$ takes into account the fact that transmission is lower near the margins of the day because of haze in the morning and clouds in the afternoon.

Following the empirical algorithm of Goudriaan and van Laar (1994), the fraction of diffuse radiation, f_d, is a function of the atmospheric transmissivity, τ:

$$f_d = \begin{cases} 1 & \tau \le 0.22 \\ 1 - 0.64(\tau - 0.22)^2 & 0.22 < \tau \le 0.35 \\ 1.47 - 1.66\tau & \tau > 0.35 \end{cases} \qquad \text{(C10)}$$

The atmospheric transmissivity, τ, in eqn (C10) is calculated according to eqn (C1), where the value of S_o at a particular time of the day is estimated from eqn (D1) in Appendix D. However, the value of f_d is not allowed to be lower than $0.15 + 0.85(1 - e^{-0.1/\sin\beta})$ (Goudriaan and van Laar 1994). The fraction of the direct-beam component of incoming radiation is $f_b = 1 - f_d$.

The model for photosynthesis (described in the main text) requires quantum flux density for PAR. A simple conversion factor of 4.56 μmol PAR J^{-1}PAR was used in the calculations.

Appendix D *Time course of solar radiation and air temperature*

Following Goudriaan and van Laar (1994), the instantaneous global radiation at a particular time of the daytime period, S_o, is estimated from the daily global radiation (S):

$$S_o = S \sin\beta(1 + 0.4\sin\beta) / D_{\sin\beta e} \qquad \text{(D1)}$$

where $\sin\beta$ and $D_{\sin\beta e}$ are given in eqn (C3) and eqn (C9), respectively.

The air temperature between sunrise and sunset was calculated from daily maximum (T_{max}) and minimum (T_{min}) temperature as (Goudriaan and van Laar 1994)

$$T_a = T_{min} + (T_{max} - T_{min})\sin[\pi(t_h + D_{la}/2 - 12)/(D_{la} + 2m)] \qquad \text{(D2)}$$

where m is the number of hours between solar noon and the time of maximum temperature (the default value for m is 1.5 hours).

To obtain the time course of temperature for a whole day, an additional equation is required for the night period (from sunset to sunrise of the next day). Goudriaan and van Laar (1994) showed an exponential equation for doing that. To implement this exponential equation together with eqn (D2), reading of weather data for consecutive days is required. For time course of temperature for a whole day used by the phenological model [eqn (46)], a simpler approach is used to derive the diurnal course of temperature using daily maximum and minimum temperature (Matthews and Hunt 1994):

$$T = 0.5\{(T_{max} + T_{min}) + (T_{max} - T_{min})\cos[\pi(i - 8)/12]\} \qquad \text{(D3)}$$

where i is the number of hours of a day (i = 1, 2, …, 24), starting with 1 for the hour of 07:00.

List of symbols (with units) used in this supplementary text 6 describing crop model GECROS

a	eqn (C4) (-)
b	eqn (C4) (-)
B_z	Stephen-Boltzmann constant (J m^{-2} s^{-1} K^{-4}) = 5.668×10^{-8}
c_{fix}	carbon cost of nitrogen fixation (g C g^{-1}N)
c_t	curvature factor in eqn (46) (-)
C	total carbon in living materials of crop (g C m^{-2}ground)
C_{max}	maximum amount of carbon in stem or seed at the end of its growth (g C m^{-2}ground)
C_R	living root carbon (g C m^{-2}ground)
C_S	living shoot carbon (g C m^{-2}ground)
$C_{\vartheta i}$	carbon demand for the growth of an organ (stem, or seed) (g C m^{-2}ground d^{-1})

D rooting depth (cm)
D_a water vapour pressure saturation deficit of air (kPa)
D_{al} air-to-leaf humidity deficit (kPa)
D_{la} astronomic daylength (h)
D_{lp} daylength for photoperiodic response of phenology (h)
D_{max} maximum rooting depth (cm)
$D_{\sin\beta}$ integral of $\sin\beta$ over the day (s d^{-1})
$D_{\sin\beta e}$ $D_{\sin\beta}$ with correction for lower atmospheric transmission at lower solar elevation (s d^{-1})
$e_{s(T_a)}$ saturated vapour pressure of air (kPa)
$e_{s(T_l)}$ saturated vapour pressure of leaf (kPa)
E_a leaf evaporation in the presence of water stress (mm s^{-1})
E_p potential leaf evaporation (mm s^{-1})
f_b fraction of direct-beam component in incoming radiation (-)
f_{car} fraction of carbohydrates in biomass (g g^{-1})
f_{clear} factor for effect of sky clearness on $R^{\uparrow}$ (-)
f_{cyc} fraction of cyclic electron transport around photosystem I (-)
$f_{c,S}$ fraction of carbon in seed biomass(g C g^{-1}dw)
$f_{c,V}$ fraction of carbon in vegetative-organ biomass (g C g^{-1}dw)
f_d fraction of diffuse component in incoming radiation (-)
f_{lig} fraction of lignins in biomass (g g^{-1})
f_{lip} fraction of lipids in biomass (g g^{-1})
f_{oac} fraction of organic acids in biomass (g g^{-1})
f_{pro} fraction of proteins in biomass (g g^{-1})
f_R ratio of root carbon to total carbon (g g^{-1})
f_S ratio of shoot carbon to total carbon (g g^{-1})
f_{vap} factor for effect of vapour pressure on $R^{\uparrow}$ (-)
g_{bc} total boundary-layer conductance in canopy (m s^{-1})
$g_{bc,sh}$ boundary-layer conductance for shaded fraction of canopy (m s^{-1})
$g_{bc,su}$ boundary-layer conductance for sunlit fraction of canopy (m s^{-1})
$g_{c,p}$ potential conductance for CO_2 (m s^{-1})
$g(T)$ function for phenological response to temperature (-)
G_w(i) normalized Gaussian weights (-)
G_x(i) normalized Gaussian distances (-)
$h(D_{lp})$ function for phenological response to photoperiod (-)
H plant height (m)
H_{max} maximum plant height (m)
I leaf absorbed photosynthetically active radiance (PAR) (μmol m^{-2}leaf s^{-1})
I_{b0} incident direct-beam radiation above canopy (J m^{-2}ground s^{-1})
I_c absorbed total radiation by canopy (J m^{-2}ground s^{-1})
$I_{c,sh}$ absorbed total radiation by shaded leaves of canopy (J m^{-2}ground s^{-1})
$I_{c,su}$ absorbed total radiation by sunlit leaves of canopy (J m^{-2}ground s^{-1})
I_{d0} incident diffuse radiation above canopy (J m^{-2}ground s^{-1})

k_b direct-beam radiation extinction coefficient (m^2ground m^{-2}leaf)
k_b' scattered-beam radiation extinction coefficient (m^2ground m^{-2}leaf)
k_d' diffuse radiation extinction coefficient (m^2ground m^{-2}leaf)
k_n nitrogen extinction coefficient (m^2ground m^{-2}leaf)
k_r PAR extinction coefficient (m^2ground m^{-2}leaf)
k_R extinction coefficient of root weight density (cm^{-1})
k_{Rn} extinction coefficient of root nitrogen concentration (m^2ground g^{-1}dw)
k_w wind-speed extinction coefficient (m^2ground m^{-2}leaf)
L green leaf area index of canopy (m^2leaf m^{-2}ground)
L_C carbon-determined L (m^2leaf m^{-2}ground)
L_i L counted from the top to the i-th layer of canopy (m^2leaf m^{-2}ground)
L_N nitrogen-determined L (m^2leaf m^{-2}ground)
L_T total (green + senesced) leaf area index (m^2leaf m^{-2}ground)
m number of hours between noon and time of maximum temperature (h) = 3
m_R minimum number of days for seed filling phase (d)
m_V minimum number of days for vegetative growth phase (d)
M_{op} maximum or minimum optimum photoperiod (h)
n_0 canopy upper leaf nitrogen (g N m^{-2}leaf)
n_{act} actual shoot nitrogen concentration (g N g^{-1}dw)
n_b minimum leaf nitrogen for photosynthesis (g N m^{-2}leaf)
n_{bot} canopy bottom leaf nitrogen (g N m^{-2}leaf)
n_{botE} n_{bot} calculated from exponential nitrogen profile (g N m^{-2}leaf)
n_{cri} critical shoot nitrogen concentration (g N g^{-1}dw)
n_{cri0} initial critical shoot nitrogen concentration (g N g^{-1}dw)
n_L average nitrogen concentration in leaf (g N g^{-1}dw)
n_{Lmin} minimum nitrogen concentration in leaf (g N g^{-1}dw)
n_{Rmin} minimum nitrogen concentration in root (g N g^{-1}dw)
n_{RV} nitrogen concentration in reserves (g N g^{-1} reserves)
n_{Smin} minimum nitrogen concentration in stem (g N g^{-1}dw)
n_{SO} standard nitrogen concentration in seed (g N g^{-1}dw)
N_c total photosynthetically effective leaf nitrogen in canopy (g N m^{-2}ground)
$N_{c,sh}$ photosynthetically effective nitrogen in shaded leaves of canopy (g N m^{-2}ground)
$N_{c,su}$ photosynthetically effective nitrogen in sunlit leaves of canopy (g N m^{-2}ground)
N_{dem} crop nitrogen demand (g N m^{-2}ground d^{-1})
N_{demA} activity-driven crop nitrogen demand (g N m^{-2}ground d^{-1})
N_{demD} deficiency-driven crop nitrogen demand (g N m^{-2}ground d^{-1})
N_{fix} symbiotically fixed nitrogen (g N m^{-2}ground d^{-1})
N_{fixD} crop demand-determined N_{fix} (g N m^{-2}ground d^{-1})
N_{fixE} available energy-determined N_{fix} (g N m^{-2}ground d^{-1})
N_{LV} living leaf nitrogen in canopy (g N m^{-2}ground)
N_{maxup} maximum crop nitrogen uptake (g N m^{-2}ground d^{-1})
N_R living root nitrogen (g N m^{-2}ground)

N_S	living shoot nitrogen (g N m^{-2}ground)
N_{SR}	living structural root nitrogen (g N m^{-2}ground)
N_T	total nitrogen in living part of the whole crop (g N m^{-2}ground)
N_{upt}	crop nitrogen uptake (g N m^{-2}ground d^{-1})
N_{uptA}	crop ammonium-nitrogen uptake (g N m^{-2}ground d^{-1})
N_{uptN}	crop nitrate-nitrogen uptake (g N m^{-2}ground d^{-1})
O_{av}	average projection of leaves in the direction of solar beam (m^2ground m^{-2}leaf)
O_{Nfix}	reserve pool of fixed nitrogen (g N m^{-2}ground)
p_{sen}	photoperiod sensitivity of phenological development (h^{-1})
P_a	gross leaf photosynthesis (g CO_2 m^{-2}leaf s^{-1})
P_C	daily gross canopy photosynthesis (g CO_2 m^{-2}ground d^{-1})
P_C(i)	instantaneous gross canopy photosynthesis (g CO_2 m^{-2}ground s^{-1})
q	fraction or ratio of minimum to standard seed N concentration (-)
r_{bh}	leaf boundary-layer resistance to heat (s m^{-1})
r_{bw}	leaf boundary-layer resistance to water (s m^{-1})
r_{LV}	relative leaf death rate (d^{-1})
$r_{sw,a}$	leaf stomatal resistance to water in the presence of water stress (s m^{-1})
$r_{sw,p}$	leaf stomatal resistance to water in the absence of water stress (s m^{-1})
r_t	turbulent resistance (s m^{-1})
R_{ng}	non-growth components of respiration (g C m^{-2}ground d^{-1})
R_{ngx}	as R_{ng}, but excluding the cost by nitrogen fixation (g C m^{-2}ground d^{-1})
R_{rmr}	residual maintenance respiration (g C m^{-2}ground d^{-1})
R_n	net radiation absorbed by leaf (J m^{-2}leaf s^{-1})
$R^{\uparrow}$	outgoing long wave radiation (J m^{-2}leaf s^{-1})
s	slope of the curve relating saturation vapour pressure to temperature (kPa°C^{-1})
s_{la}	specific leaf area constant for new leaves (m^2leaf g^{-1}leaf)
S	daily global radiation (J m^{-2} d^{-1})
S_c	solar constant (J m^{-2} s^{-1})
S_f	number of seeds to be filled (seeds m^{-2}ground)
S_o	radiation incident on a horizontal earth surface (J m^{-2} s^{-1})
S_w	expected weight of single seed (g $seed^{-1}$)
t_d	day of the year (d)
t_e	development stage for the end of seed-number determining period (-)
t_h	time of the day (h)
T	diurnal temperature in eqn (46) (°C)
T_a	daytime air temperature (°C)
T_b	base temperature for phenological development (°C)
T_c	ceiling temperature for phenological development (°C)
TC_s	time constant for senescence (d)
T_l	daytime leaf temperature (°C)
T_{max}	daily maximum air temperature (°C)
T_{min}	daily minimum air temperature (°C)
T_o	optimum temperature for phenological development (°C)

u wind speed (m s^{-1})
V vapour pressure (kPa)
w leaf blade width (m)
W_{LV} living leaf weight (g dw m^{-2}ground)
W_R living root weight (g dw m^{-2}ground)
W_{RT} total root weight (g dw m^{-2}ground)
W_S living shoot weight (g dw m^{-2}ground)
W_{SR} living structural root weight (g dw m^{-2}ground)
$W_{SR,N}$ nitrogen-determined W_{SR} (g dw m^{-2}ground)
W_T living crop weight (g dw m^{-2}ground)
Y_G growth efficiency (g C g^{-1}C)
$Y_{G,S}$ storage-organ (seed) growth efficiency (g C g^{-1}C)
$Y_{G,V}$ vegetative-organ (leaf, stem, root) growth efficiency (g C g^{-1}C)
α sun angle below horizon to calculate D_{lp} (degrees)
β solar elevation (degrees)
β_L leaf inclination angle in canopy (degrees)
δ declination of the sun (radians)
ΔC_{LV} rate of change in living leaf carbon (g C m^{-2}ground d^{-1})
ΔL_C rate of change of L_C (m^2leaf m^{-2}ground d^{-1})
Δn_{bot} rate of change of n_{bot} (g N m^{-2}leaf d^{-1})
ΔN_{LV} rate of change of N_{LV} (g N m^{-2}ground d^{-1})
ΔN_{LV}^- loss rate of leaf nitrogen due to senescence (g N m^{-2}ground d^{-1})
ΔN_R^- loss rate of root nitrogen due to senescence (g N m^{-2}ground d^{-1})
Δt time step of dynamic simulation (d)
ΔT leaf-to-air temperature differential (°C)
ΔW_{LV}^- rate of leaf senescence (g dw m^{-2}ground d^{-1})
ΔW_{RT} rate of change in total root weight (g dw m^{-2}ground d^{-1})
ΔW_R^- rate of root senescence (g dw m^{-2}ground d^{-1})
ε_g germination efficiency, i.e. dry weight of seedling to per g dry seed (g dw g^{-1}dw)
ϕ_{sh} fraction of shaded leaves in a canopy (-)
ϕ_{su} fraction of sunlit leaves in a canopy (-)
$\phi_{su,i}$ fraction of sunlit leaves at canopy depth L_i (-)
γ Psychrometric constant (kPa $°C^{-1}$) = 0.067
η eqn (32) (g N g^{-1}C)
ϑ development stage (-)
ϑ_1 development stage at which plant starts to become sensitive to photoperiod (-)
ϑ_2 development stage at which plant ends to respond to photoperiod (-)
ϑ_e development stage at the end of growth of stem or seed (-)
ϑ_i development stage during the growth of stem or seed (-)
ϑ_m development stage at the time of maximal growth rate of stem or seed (-)
κ nitrogen-carbon ratio in crop (g N g^{-1}C)

λ latent heat of vaporization of water vapour (J kg^{-1}water) = 2.4×10^{6}
$\lambda_{C,leaf}$ fraction of newly assimilated shoot carbon partitioned to leaf (g C g^{-1}C)
$\lambda_{C,seed}$ fraction of newly assimilated shoot carbon partitioned to seed (g C g^{-1}C)
$\lambda_{C,stem}$ fraction of newly assimilated shoot carbon partitioned to stem (g C g^{-1}C)
$\lambda_{C,Sres}$ fraction of newly assimilated shoot carbon partitioned to stem reserve pool (g C g^{-1}C)
$\lambda_{C,S}$ fraction of newly assimilated carbon partitioned to shoot (g C g^{-1}C)
$\lambda_{N,S}$ fraction of newly absorbed nitrogen partitioned to shoot (g C g^{-1}C)
ρ proportion factor between stem biomass and plant height (g dw m^{-1})
ρ_{cb} canopy beam radiation reflection coefficient (-)
ρ_{cd} canopy diffuse radiation reflection coefficient (-)
ρ_{h} canopy reflection coefficient for horizontal leaves (-)
ρc_{p} volumetric heat capacity of air (J m^{-3} °C^{-1}) = 1200
σ leaf scattering coefficient (-) = 0.2 for PAR, and 0.8 for NIR
σ_{C} relative shoot activity (g C g^{-1}C d^{-1})
σ_{N} relative root activity (g N g^{-1}C d^{-1})
τ atmospheric transmissity (-)
τ_{C} time constant in eqns (28) and (29) (d)
υ_{C0} ratio of initial shoot carbon or biomass to initial total carbon or biomass (g g^{-1})
υ_{N0} ratio of initial shoot N to initial total N (g N g^{-1}N)
ϖ specific rate of maintenance respiration (g C g^{-1}N d^{-1})
ω_{i} development rate (d^{-1})
ζ latitude (degrees)

References used in Supplementary Text 6 not already cited in main text

Amthor, J.S., 2000. The McCree-de Wit-Penning de Vries-Thornley respiration paradigms: 30 years later. Annals of Botany 86: 1-20.

Anten, N.P.R., 1997. Modelling canopy photosynthesis using parameters determined from simple non-destructive measurements. Ecological Research 12: 77-88.

Biscoe, P.V., Gallagher, J.N., Littleton, E.J., Monteith, J.L. and Scott, R.K., 1975. Barley and its environment. IV. Sources of assimilate for the grain. Journal of Applied Ecology 12: 295-318.

Bradbury, N.J., Whitmore, A.P., Hart, P.B.S. and Jenkinson, D.S., 1993. Modelling the fate of nitrogen in crop and soil in the years following application of ^{15}N-labelled fertilizer to winter wheat. Journal of Agricultural Science, Camb. 121: 363-379.

Brouwer, R., 1983. Functional equilibrium: sense or nonsense? Netherlands Journal of Agricultural Science 31: 335-348.

Cannell, M.G.R. and Thornley, J.H.M., 2000. Modelling the components of plant respiration: Some guiding principles. Annals of Botany 85: 45-54.

Charles-Edwards, D.A., 1976. Shoot and root activities during steady-state plant growth. Annals of Botany 40: 767-772.

Chaves, M.M., 1991. Effects of water deficits on carbon assimilation. Journal of Experimental Botany 42: 1-16.

Cornic, G., 2000. Drought stress inhibits photosynthesis by decreasing stomatal aperture – not by affecting ATP synthesis. Trends in Plant Science 5: 187-188.

de Pury, D.G.G. and Farquhar, G.D., 1997. Simple scaling of photosynthesis from leaves to canopies without the errors of big-leaf models. Plant, Cell and Environment 20: 537-557.

Forrester, J.W., 1961. Industrial dynamics. MIT Press, Boston, USA.

Gifford, R.M., 2003. Plant respiration in productivity models: conceptualisation, representation and issues for global terrestrial carbon-cycle research. Functional Plant Biology 30: 171-186.

Godwin, D.C. and Jones, C.A., 1991. Nitrogen dynamics in soil-plant systems. P.287-321. In: J. Hanks and J.T. Ritchie (editors) Modelling Plant and Soil Systems – Agronomy Monograph no. 31. American Society of Agronomy, Madison, Wisconsin.

Goudriaan, J., 1977. Crop Micrometeorology – a Simulation Study. Simulation Monographs, Pudoc, Wageningen.

Goudriaan, J. 1982. Potential production processes. P. 98-113. In: F.W.T. Penning de Vries and H. H. van Laar (eds), Simulation of plant growth and crop production. Pudoc, Wageningen.

Goudriaan, J., 1986. A simple and fast numerical method for the computation of daily totals of crop photosynthesis. Agricultural and Forest Meteorology 38: 249-254.

Goudriaan, J., 1988. The bare bones of leaf-angle distribution in radiation models for canopy photosynthesis and energy exchange. Agricultural and Forest Meteorology 43: 155-169.

Goudriaan, J. and van Laar, H.H., 1994. Modelling potential crop growth processes. Kluwer Academic Publishers, Dordrecht, The Netherlands.

Goudriaan, J., van Laar, H.H., van Keulen, H. and Louwerse, W., 1984. Simulation of the effect of increased atmospheric CO_2 on assimilation and transpiration of a closed crop canopy. Wissenschaftliche Zeitschrift der Humboldt-Universitat zu Berlin, Math.-Nat. R. 33: 352-356.

Hilbert, D.W., 1990. Optimization of plant root:shoot ratios and internal nitrogen concentration. Annals of Botany 66: 91-99.

Justes, E., Mary, B., Meynard, J.-M., Machet, J.-M. and Thelier-Huche, L., 1994. Determination of a critical nitrogen dilution curve for winter wheat crops. Annals of Botany 74: 397-407.

Leuning, R., Kelliher, F.M., De Pury, D.G.G. and Schulze, E.-D., 1995. Leaf nitrogen, photosynthesis, conductance and transpiration: scaling from leaves to canopies. Plant, Cell and Environment 18: 1183-1200.

Matthews, R.B. and Hunt,, L.A., 1994. A model describing the growth of cassava (*Manihot esculenta* L. Crantz). Field Crop Research 36: 729-739.

McArthur, A.J., 1990. An accurate solution to the Penman equation. Agricultural and Forest Meteorology 51: 87-92.

Monteith, J.L. 1973. Principles of environmental physics. Edward Arnold, London.

Peng, S. and Cassman, K.G., 1998. Upper thresholds of nitrogen uptake rates and associated nitrogen fertilizer efficiencies in irrigated rice. Agronomy Journal 90: 178-185.

Penning de Vries, F.W.T., Jansen, D.M., ten Berge, H.F.M. and Bakema, A., 1989. Simulation of ecophysiological processes of growth in several annual crops. IRRI, Los Banos and Pudoc, Wageningen.

Robertson, M.J., Carberry, P.S., Huth, N.I., Turpin, J.E., Probert, M.E., Poulton, P.L., Bell, M., Wright, G.C., Yeates, S.J. and Brinsmead, R.B., 2002. Simulation of growth and development of diverse legume species in APSIM. Australian Journal of Agricultural Research 53: 429-446.

Ryan, M.G. 1991. Effects of climate change on plant respiration. Ecological Applications 1: 157-167.

Sinclair, T.R. and De Wit, C.T., 1975. Photosynthate and nitrogen requirements for seed production by various crops. Science 189: 565-567.

Spitters, C.J.T., 1986. Separating the diffuse and direct component of global radiation and its implications for modelling canopy photosynthesis. Part II. Calculation of canopy photosynthesis. Agricultural and Forest Meteorology 38: 231-242.

van Keulen, H. and Seligman, N.G. 1987. Simulation of water use, nitrogen nutrition and growth of a spring wheat crop. Pudoc, Wageningen.

van Kraalingen, D.W.G., Rappoldt, C. and van Laar, H.H., 2003. The FORTRAN simulation translator, a simulation language. European Journal of Agronomy 18: 259-361.

van Laar, H.H., Goudriaan, J. and van Keulen, H., 1997. SUCROS97: Simulation of crop growth for potential and water-limited production situations, as applied to spring wheat. Report series No. 14, Quantitative Approaches in Systems Analysis, Wageningen.

Wang, Y.P. and Leuning, R., 1998. A two-leaf model for canopy conductance, photosynthesis and partitioning of available energy. I. Model description and comparison with a multi-layered model. Agricultural and Forest Meteorology 91: 89-111.

Weiland, R.T., Stutte, C.A. and Silva, P.R.F., 1982. Nitrogen volatilization from plant foliage. Report series No 266, Agricultural Experiment Station, University of Arkansas, 40pp.

Wilson, J.B., 1988. A review of evidence on the control of shoot : root ratio, in relation to models. Annals of Botany 61: 433-449.

Yin, X. and Schapendonk, A.H.C.M., 2004. Simulating the partitioning of biomass and nitrogen between root and shoot in crop and grass plants. NJAS-Wageningen Journal of Life Sciences 51: 407-426.

Yin, X. and van Laar, H.H., 2005. Crop Systems Dynamics: An Ecophysiological Simulation Model for Genotype-by-Environment Interactions. Wageningen Academic Publishers, Wageningen, The Netherlands, 155pp (ISBN9076998558).

Yin, X., Kropff, M.J., McLaren, G. and Visperas, R.M., 1995. A nonlinear model for crop development as a function of temperature. Agricultural and Forest Meteorology 77: 1-16.

Yin, X., Goudriaan, J., Lantinga, E.A., Vos, J. and Spiertz, J.H.J., 2003a. A flexible sigmoid function of determinate growth. Annals of Botany 91: 361-371 (erratum in Annals of Botany 91: 753, 2003).

Yin, X., Lantinga, E.A., Schapendonk, A.H.C.M. and Zhong, X., 2003b. Some quantitative relationships between leaf area index and canopy nitrogen content and distribution. Annals of Botany 91: 893-903.

Yin, X., Schapendonk, A.H.C.M., Kropff, M.J., van Oijen, M. and Bindraban, P.S., 2000. A generic equation for nitrogen-limited leaf area index and its application in crop growth models for predicting leaf senescence. Annals of Botany 85: 579-585.
